# Characterization and induction of prophages in human gut-associated *Bifidobacterium* hosts

**DOI:** 10.1038/s41598-018-31181-3

**Published:** 2018-08-24

**Authors:** Travis N. Mavrich, Eoghan Casey, Joana Oliveira, Francesca Bottacini, Kieran James, Charles M. A. P. Franz, Gabriele Andrea Lugli, Horst Neve, Marco Ventura, Graham F. Hatfull, Jennifer Mahony, Douwe van Sinderen

**Affiliations:** 1 School of Microbiology, University College Cork, Cork, Ireland; 2 APC Microbiome Ireland, University College Cork, Cork, Ireland; 3 Department of Biological Sciences, University of Pittsburgh, Pittsburgh, PA 15260 USA; 4 Department of Microbiology and Biotechnology Max Rubner-Institut, Kiel, Germany; 5 Laboratory of Probiogenomics, Department of Chemistry, Life Sciences and Environmental Sustainability, University of Parma, Parma, Italy

## Abstract

In the current report, we describe the identification of three genetically distinct groups of prophages integrated into three different chromosomal sites of human gut-associated *Bifidobacterium breve* and *Bifidobacterium longum* strains. These bifidobacterial prophages are distantly related to temperate actinobacteriophages of several hosts. Some prophages, integrated within the *dnaJ*_*2*_ gene, are competent for induction, excision, replication, assembly and lysis, suggesting that they are fully functional and can generate infectious particles, even though permissive hosts have not yet been identified. Interestingly, several of these phages harbor a putative phase variation shufflon (the Rin system) that generates variation of the tail-associated receptor binding protein (RBP). Unlike the analogous coliphage-associated shufflon Min, or simpler Cin and Gin inversion systems, Rin is predicted to use a tyrosine recombinase to promote inversion, the first reported phage-encoded tyrosine-family DNA invertase. The identification of bifidobacterial prophages with RBP diversification systems that are competent for assembly and lysis, yet fail to propagate lytically under laboratory conditions, suggests dynamic evolution of bifidobacteria and their phages in the human gut.

## Introduction

Bacteriophages are the most abundant biological entities^1^ and exhibit incredible genetic diversity^2^. By impacting the growth and evolution of their bacterial hosts, they play powerful roles in their environment^3^, such as the human gut microbiome. This dynamic microbial community is comprised of hundreds of species spanning numerous phyla and genera^4^, and their complex interactions are believed to influence human health^5^. Phages are abundant in this environment^6,7^ and can be used to artificially modulate the community^8^.

Bacteria of the genus *Bifidobacterium* are prevalent and important members of the gut^9^. These organisms are Gram-positive and anaerobic, and represent members of the phylum Actinobacteria. Bifidobacteria are the dominant bacterial taxon that starts populating the gut immediately after birth, that persists across the human lifespan, and that is associated with eliciting a positive host health status^9^. Two of the most abundant bifidobacterial species in the infant gut are *Bifidobacterium breve* and *Bifidobacterium longum*^10^. Study and manipulation of these two species using bifidobacterial phages (bifidophages) would enhance our understanding of the gut microbiome. However, isolation and propagation procedures for bifidophages infecting these species have to our knowledge not yet been reported.

In contrast to the lack of (propagating) phages for these hosts, phages infecting other hosts in the phylum Actinobacteria (actinobacteriophages) are readily isolated^11^. Currently, there are over 2,500 sequenced actinobacteriophages infecting hosts from over 10 genera, the majority of which infect a single genus, *Mycobacterium*, being grouped into clusters based on gene content and sequence similarity^11^. Expanding the phylogenetic breadth of host genera, such as *Propionibacterium*^12^, *Arthrobacter*^13^, and *Gordonia*^14^, has enabled comparative analyses that continually enhance our understanding of phage biology, diversity, and host interactions. The lack of characterized bifidophages limits further exploration of actinobacteriophage biology in general, and the investigation of their role in the human gut in particular.

Comparisons of bifidobacterial genomes suggest that bifidophages are abundant. Efforts to characterize bifidobacterial diversity in the human gut have resulted in numerous sequenced *B. breve* and *B. longum* isolates^15–18^. The majority of these strains are predicted to contain at least one complete or cryptic prophage^19,20^, such as prophages Binf-1 from *B. longum* subsp*. infantis* ATCC 15697^21^ and 689b-1 from *B. breve* 689b^16^, or the cryptic prophage Bbr-1 in *B. breve* UCC2003^22^. Genetically related prophages are present in multiple bifidobacterial species, indicating that they have either broad or dynamic host range specificities. Many strains contain phage defense strategies such as restriction-modification systems^18^ or CRISPR arrays with spacers that match many of the predicted prophages^21,23^, the latter suggesting frequent host-phage interactions. In addition, excision of some prophages can be induced by mitomycin C, as recently reported for those in the uncharacterized bifidobacterial species, *B. choerinum* LMG 10510^15^ and *B. moukalabense* DSM 27321^24^, which produces apparently complete phage particles^19^. However, there have been no reports of inducible phage particles from bifidobacterial strains that are more closely associated with the human gut.

Here, we identified three groups of related prophages in several *B. breve* and *B. longum* strains. These prophages are distantly related to several types of actinobacteriophages of other hosts, and they are either integrated into a tmRNA gene, a tRNA^Met^ gene, or the *dnaJ*_*2*_ gene, the latter representing an atypical phage integration site that appears to be unique to bifidobacteria/Actinobacteria. We successfully induce some of the *dnaJ*_*2*_-integrated prophages using mitomycin C and show that they replicate and assemble into complete phage particles. Many of them contain a putative tyrosine DNA invertase-mediated phase variation shufflon, Rin, that modulates the receptor binding protein (RBP) allele, analogous to the coliphage Min shufflon.

## Results and Discussion

### Identification of *B. breve* and *B. longum* prophages

**Figure 1:**
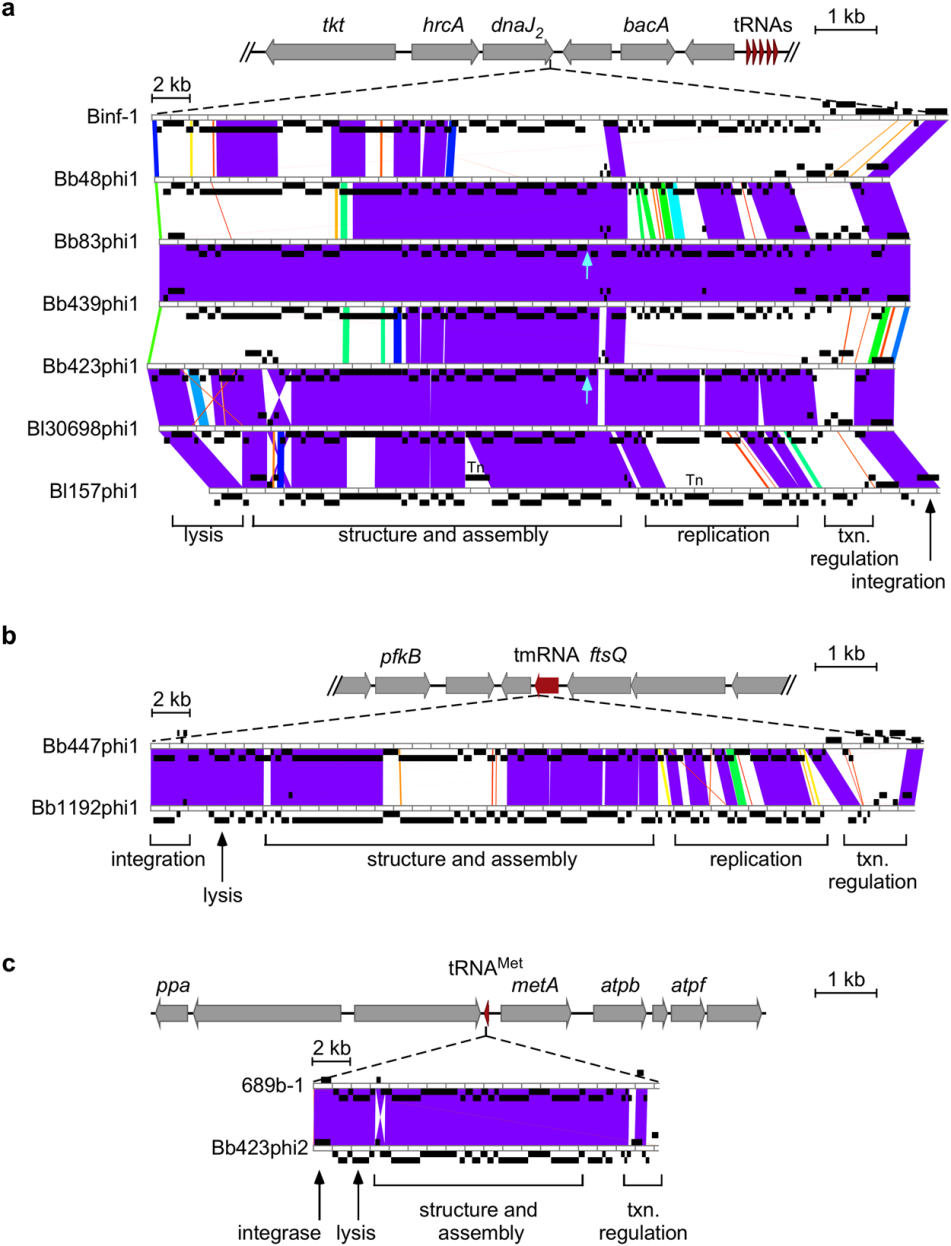
Bifidoprophage genomic comparison and characterization. (**a**) (top) Enlarged view of the *dnaJ*_*2*_ integration locus. Coding (grey) and tRNA (red) genes are indicated, oriented in the direction of transcription, and with gene descriptions indicated where applicable. The seven prophages are integrated at the 3′ end of the *dnaJ*_*2*_ gene. (bottom) Genome architecture and mosaic relationships between the seven prophage genomes are highlighted with pairwise alignments in Phamerator. Genes (black boxes) are positioned above or below the genome ruler to indicate orientation. The color spectrum between genomes reflects sequence similarity based on BLAST e-values, ranging from white (no similarity) to violet (high similarity). Cyan arrows indicate area of lowest sequencing coverage from the induced virion genome indicating the location of the linear virion genome termini. General regions of specific gene modules are indicated below the alignment. Tn = transposase. (**b**) (top) Enlarged view of the tmRNA integration locus, as in panel (a). The prophages are integrated at the 3′ end of the tmRNA gene. (bottom) Genome architecture and mosaic relationships between the prophage genomes, as in panel (a). (**c**) (top) Enlarged view of the tRNA^Met^ integration locus, as in panel (a). The prophages are integrated at the 3′ end of the tRNA^Met^ gene. (bottom) Genome architecture and mosaic relationships between the prophage genomes, as in panel (a). Note: in each panel, the host and prophage genome maps are on different scales.

Although prophages have been predicted in several *Bifidobacterium* strains, many are not (or are at best poorly) characterized, and it is not known if they retain the functional capacity to form infectious particles. To investigate this, we first genomically defined several predicted prophages and compared them against known phage genomes. We examined a total of eleven prophages present in seven *B. breve* and three *B. longum* strains (Fig. 1, Tables 1, 2). Five *B. breve* isolates (082W4–8; 180W8-3; 139W4-23; 017W4–39; 215W4–47a) were recently reported to harbor six prophages^18^, designated here as Bb48phi1, Bb83phi1, Bb423phi1, Bb423phi2, Bb439phi1, and Bb447phi1. BLAST homology searches using prophage-encoded integrase genes identified five additional prophages in five other strains, including *B. breve* JCM 1192, *B. breve* 689b, *B. longum* subsp. *infantis* ATCC 15697^25^, *B. longum* subsp. *longum* 157F^25^, and *B. longum* subsp. *longum* CCUG 30698^17^. Two of these prophages, 689b-1^16^ and Binf-1^21^, have been noted previously, while the other three, Bb1192phi1, Bl157phi1, and Bl30698phi1, are newly identified.

**Table 1:** *Bifidobacterium* genomes used in this study.

Strain	Accession	Reference	Strain type (for data analysis)	GC%
*B. choerinum* LMG 10510	JGYU00000000	Milani *et al*.^15^	Lysogen	65.5
*B. moukalabense* DSM 27321	AZMV01000000	Lugli *et al*.^24^	Lysogen	59.9
*B. longum infantis* ATCC 15697	AP010889	Fukuda *et al*.^25^	Lysogen	59.9
*B. breve* JCM 7017	CP006712	Bottacini *et al*.^16^	Non-lysogen	58.7
*B. breve* NCIMB 702258	CP006714	Bottacini *et al*.^16^	Non-lysogen	58.7
*B. breve* UCC2003	CP000303	Motherway *et al*., 2011	Non-lysogen	58.7
*B. breve* 082W4–8	CP021555	Bottacini *et al*.^18^	Predicted lysogen	58.8
*B. breve* 180W8–3	CP021557	Bottacini *et al*.^18^	Predicted lysogen	58.8
*B. breve* 139W4–23	CP021556	Bottacini *et al*.^18^	Predicted lysogen	58.6
*B. breve* 017W4–39	CP021554	Bottacini *et al*.^18^	Predicted lysogen	58.7
*B. breve* 215W4–47a	CP021558	Bottacini *et al*.^18^	Predicted lysogen	59.3
*B. longum longum* CCUG 30698	CP011965	O’Callaghan *et al*.^17^	Predicted lysogen	60.2
*B. longum longum* 157F	AP010890	Fukuda *et al*.^25^	Predicted lysogen	60.1
*B. breve* JCM 1192	AP012324	*Not applicable*	Predicted lysogen	58.9
*B. breve* 689b	CP006715	Bottacini *et al*.^16^	Predicted lysogen	58.7

**Table 2:** Bifidoprophage genomes analyzed in this study.

Prophage	Host strain	Reference	Left boundary^a^	Right boundary^a^	Size (nt)	GC%	Integration Locus	Group^d^
Bb48phi1	*B. breve* 082W4–8	Bottacini *et al*.^18^	1,193,403	1,232,804	39,402	61.3	*dnaJ*_*2*_ (*BB08*2*W48_0987*)	3
Bb83phi1	*B. breve* 180W8–3	Bottacini *et al*.^18^	1,179,127	1,219,381	40,255	61.1	*dnaJ*_*2*_ (*BB180W83_0986*)	3
Bb423phi1	*B. breve* 139W4–23	Bottacini *et al*.^18^	1,302,639	1,342,715	40,077	60.9	*dnaJ*_*2*_ (*BB139W423_1102*)	3
Bb439phi1	*B. breve* 017W4–39	Bottacini *et al*.^18^	1,194,717	1,234,971	40,255	61.1	*dnaJ*_*2*_ (*BB017W439_1000*)	3
Binf-1^b^	*B. longum* *infantis* ATCC 15697	Ventura *et al*.^21^	1,288,185	1,330,866	42,682	61.1	*dnaJ*_*2*_ (*BLIJ_1123*)	3
Bl30698phi1	*B. longum longum* CCUG 30698	this study	1,375,860	1,336,483	39,378	61.1	*dnaJ*_*2*_ (*BBL306_1177*)	3
Bl157phi1	*B. longum longum* 157F	this study	1,246,936	1,207,777	39,160	60.9	*dnaJ*_*2*_ (*BLIF_1084*)	3
Bb447phi1	*B. breve* 215W4–47a	Bottacini *et al*.^18^	1,694,074	1,735,438	41,365	58.6	tmRNA^c^	4
Bb1192phi1	*B. breve* JCM 1192	this study	1,468,985	1,509,872	40,888	59.4	tmRNA^c^	4
Bb423phi2	*B. breve* 139W4–23	Bottacini *et al*.^18^	420,545	438,765	18,221	61.4	tRNA^Met^	1
689b-1	*B. breve* 689b	Bottacini *et al*.^16^	372,287	390,546	18,260	61.4	tRNA^Met^	1

^a^Coordinates based on sequence orientation in published Genbank record. For prophages integrated at *dnaJ*_*2*_ gene, left and right boundaries are based on empirically determined site of strand exchange. Otherwise, boundaries are determined based on entire attachment sites.

^b^Prophage coordinates and integration locus modified from previous description^19,21^, due to analysis with other prophages in this study.

^c^tmRNA is not annotated in this Genbank record.

^d^As determined by whole genome phylogenetic analysis in Fig. 2b.

**Figure 2:**
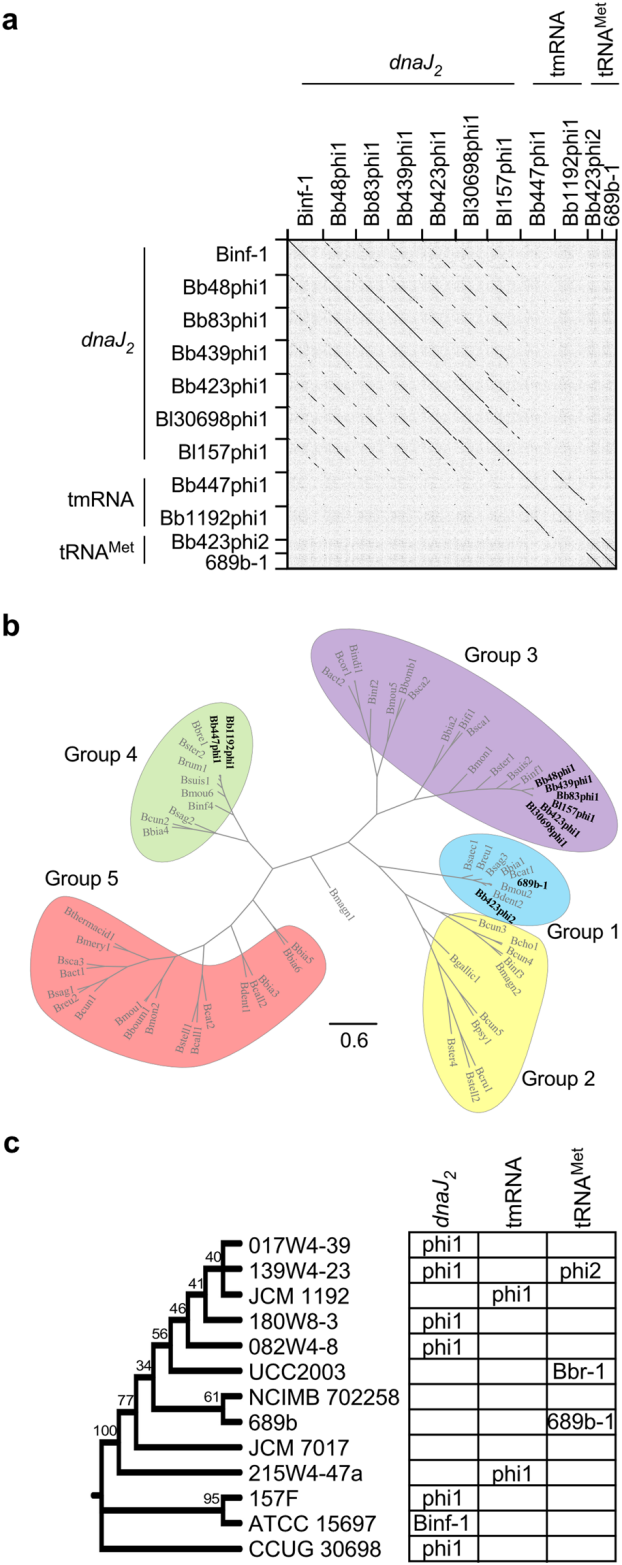
Genomic relationships of bifidoprophages and their hosts. (**a**) Gepard dotplot analysis highlights prophage pairwise sequence similarities. (**b**) Phylogenetic analysis of sixty previously reported bifidoprophages (grey)^19^ and eleven newly characterized bifidoprophages (black) using whole genome alignments and grouped as previously described^19^. (**c**) Cladogram of bifidobacterial host genomes constructed from alignment of 16S rRNA sequences; % bootstrap branch support indicated. Table indicates the presence or absence of prophages integrated at the *dnaJ*_*2*_, tmRNA, or tRNA^Met^ loci.

Genomic features of the eleven prophages were characterized and compared (salient features can be found in Tables 2 and 3). All prophages exhibit a similar GC% content as their hosts (Tables 1, 2), they are predicted to encode a tyrosine integrase, and they are integrated at (homologs of) three different loci (Fig. 1). The genome architectures of the prophages at each locus are similar, with gene modules of similar functions syntenically ordered, but with characteristically mosaic relationships. Dotplot analysis shows that the prophages integrated at (homologs of) the same locus exhibit sequence similarity to each other, yet are not closely related to prophages positioned at the other loci (Fig. 2a). Phylogenetic analysis using whole genome alignment indicates they are related to three distinct groups of previously reported bifidoprophages (Fig. 2b, Table 2)^19^. Gene function prediction using BLAST^26^ and HHpred^27^ identified many genes related to phage growth, including DNA replication, virion assembly, and prophage integration. The genomic relationships of these prophages were compared to each other and to more than 1,000 isolated, sequenced, and manually annotated actinobacteriophages using Phamerator, which identifies regions of nucleotide homology and groups genes into phamilies (phams) based on amino acid sequence relationships^28^.

**Table 3:** Bifidoprophage *attL* and *attR* common core sites.

Integration locus	Prophage	Attachment site	Sequence
*dnaJ*_*2*_	Bb48phi1	*attL*	TTCTTTAGCAAGTTAAAAGACGCACTGAGCTGAGA
		*attR*	TTCTTCAGCAAGTTAAAGGATGCCTTGAGCTGATT
	Bb83phi1	*attL*	TTCTTTAGCAAGTTGAAGGACGCACTGAGCTGAGA
		*attR*	TTCTTCAGCAAGTTAAAGGATGCCTTGAGCTGATT
	Bb423phi1	*attL*	TTCTTTAGCAAGTTGAAGGACGCGCTGAGCTGAGA
		*attR*	TTCTTCGCAAGTTAAAGGATGCCTTGAGCTGATT
	Bb439phi1	*attL*	TTCTTTAGCAAGTTGAAGGACGCACTGAGCTGAGA
		*attR*	TTCTTCAGCAAGTTAAAGGATGCCTTGAGCTGATT
	Binf-1	*attL*	TTCTTCAGCAAGTTAAAAGACGCACTGAGCTGAGA
		*attR*	TTCTTCTGCAAGTTAAAAGACGCACTGAGCTGAGA
	Bl30698phi1	*attL*	TTCTTCAGCAAGTTGAAGGACGCGCTGAGCTGAGA
		*attR*	TTCTTCGCAAGTTGAAGGACGCACTGAGCTGAGA
	Bl157phi1	*attL/attR*	TTCTTCAGCAAGTTGAAGGACGCACTGAGCTGAGA
tmRNA	Bb447phi1/Bb1192phi1	*attL/attR*	GTGGAGTCGCGGGGAATCGAACCCCG
tRNA^Met^	Bb423phi2/689b-1	*attL*	TGGTAGCGGGGCATGGATTTGAACCTTGGACCTCTGGGT
		*attR*	TGGTAGCGGGGCATGGATTTGAACCATGGACCTCTGGGT

**Figure 3:**
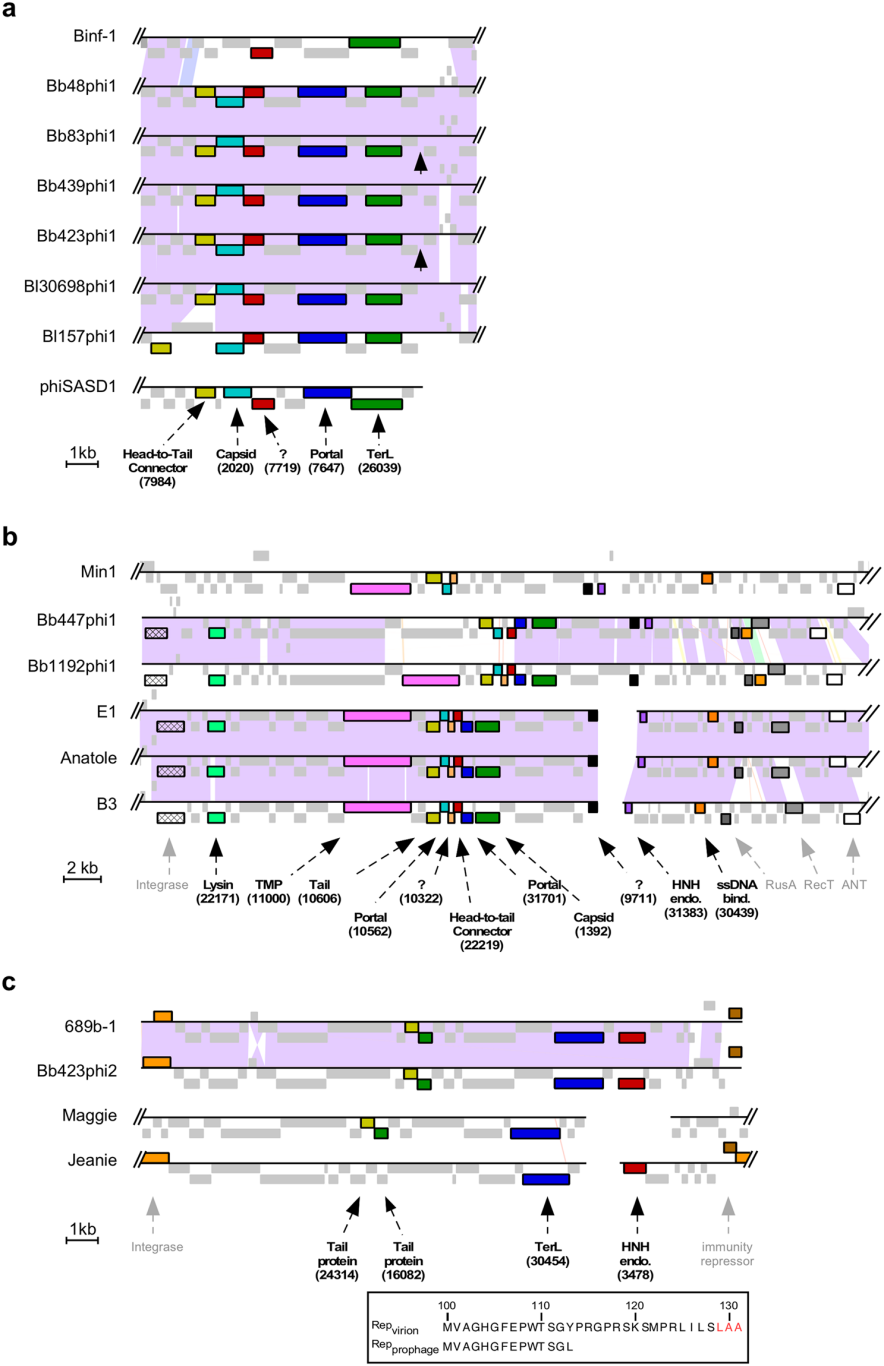
Evolutionary relationships of bifidoprophages to other actinobacteriophages. (**a**) Enlarged view of the structural gene locus of *dnaJ*_*2*_-integrated prophages, from Fig. 1a. *Streptomyces* phage phiSASD1 (in virion orientation) has been included for comparison. Genes of all phams that are shared between phiSASD1 and at least one of the *dnaJ*_*2*_-integrated prophages are highlighted. Each pham is uniquely color-coded and labeled with the predicted function and pham number. All other genes are grey. Black arrows indicate the location of the linear virion genome termini. (**b**) Enlarged view of tmRNA-integrated prophages from Fig. 1b. *Microbacterium* phage Min1 and *Propionibacterium* phages E1, Anatole, and B3 (in virion orientation) have been included for comparison. Genes of all phams that are shared between the *Propionibacterium* or *Microbacterium* phage and at least one tmRNA-integrated prophage are highlighted. Each pham is uniquely color-coded and labeled in bold with the predicted function and pham number. Genes that are present in all genomes and that have a similar predicted function, but are not in the same pham, are also highlighted. Each gene is uniquely color-coded and labeled with the function in grey, but pham numbers are omitted. All other genes are grey. (**c**) Enlarged view of tRNA^Met^-integrated prophages from Fig. 1c. *Arthrobacter* phage Maggie and *Gordonia* phage Jeanie (in virion orientation) have been included for comparison. Genes colored and indicated as in panel (b). (Inset) C-terminal amino acid sequence of the Bb423phi2 immunity repressor (Rep) translated from the sequenced prophage and predicted virion alleles, with a *ssrA*-like ClpX recognition motif highlighted (red).

Four *B. breve* prophages (Bb48phi1, Bb83phi1, Bb423phi1, and Bb439phi1), and three *B. longum* prophages (Bl157phi1, Bl30698phi1, and Binf-1) are integrated at the *dnaJ*_*2*_ locus (Fig. 1a)^22^. The *dnaJ*_*2*_ gene is one of two *dnaJ* homologs present in the *Bifidobacterium* genome. It encodes a highly conserved molecular chaperone involved in stress response, similar to its paralog *dnaJ*_1_, is only present in the Actinobacteria phylum^22^, and is reconstructed following phage integration. Integration within the coding region of the *dnaJ*_*2*_ gene by a tyrosine integrase is unusual. Tyrosine integrases typically use attachment sites overlapping tRNA or tmRNA genes, in contrast to serine integrases that more commonly use *attB* sites within coding regions^29^. The bifidoprophages possess 35 bp *attL* sites that overlap the 3′-end of the *dnaJ*_*2*_ gene, and range in size from ~39–43 kb. Two of them (Bb83phi1 and Bb439phi1) are nearly identical (Fig. 1a, Tables 2, 3). One of the prophages, Binf-1, was previously identified, although its precise integration site was not known^19,21^. Among these seven prophages, nearly 150 phams are represented, of which just 18 are present in phages of other actinobacterial hosts. The closest relative is temperate *Streptomyces* phage phiSASD1 (Fig. 3a)^30^. Although there is no significant nucleotide sequence similarity, phiSASD1 harbors five syntenically positioned genes in shared phams with these prophages, including predicted terminase, portal, capsid, and head-to-tail connector genes. Genes corresponding to the other 13 shared phams are distributed among various other actinobacteriophages.

Two *B. breve* prophages, Bb447phi1 and Bb1192phi1, are integrated at a tmRNA gene (Fig. 1b). This gene is present in nearly all bacteria and is involved in releasing stalled ribosomes during translation^31^. It is a known integration site for phages in other hosts^32^, such as for the Cluster K mycobacteriophages^33^. Bb447phi1 and Bb1192phi1 have 26 bp *attR* sites overlapping the 3’ end of the tmRNA gene, they are ~41 kb long, and encompass a total of 78 phams (Fig. 1b, Tables 2, 3). Only 18 of these phams are found in phages of other actinobacterial hosts, and based on shared gene content, the closest relatives are the temperate phages in Cluster BV infecting *Propionibacterium*^34^ and phage Min1 infecting *Microbacterium*^35^ (Fig. 3b). These three groups of phages harbor 8–11 syntenically positioned genes in shared phams, many with predicted functions related to virion structure and assembly, host lysis, and DNA replication (Fig. 3b). In contrast to the similarities observed with Min1 and Cluster BV phages, Bb447phi1 and Bb1192phi1 do not share more than three phams with any other actinobacteriophage.

Two *B. breve* prophages, Bb423phi2 and 689b-1, are integrated at a tRNA^Met^ gene adjacent to *metA* (Fig. 1c). Previously, two prophage-like elements, Bbr-1 in *B. breve* UCC2003 and Bl-1 in *B. longum* NCC2705, were reported to be integrated at this site^20^, and 689b-1 was previously described though its precise integration site was not known^16^. Bb423phi2 and 689b-1 have 39 bp *attR* sites that overlap the 3’ end of the tRNA^Met^ gene (Fig. 1c, Table 3). These two prophages are just ~18 kb long (Table 2), similar to Bl-1, and encompass a total of 30 phams, most of which are related to structure and assembly. Although both Bl-1 and 689b-1 were originally classified as cryptic prophages due to their small size, many similarly sized actinobacteriophages have been reported, including the lytic *Rhodococcus* phage RRH1^36^, lytic *Arthrobacter* phages grouped in Cluster AN^13^, and temperate *Gordonia* phages grouped in Cluster CW^14^. These are the only phages of other actinobacterial hosts that share any phams with Bb423phi2 and 689b-1 (Fig. 3c). Interestingly, the Cluster CW phages such as Maggie – which exhibit the closest sequence similarity – have regulatory systems characteristic of integration-dependent immunity systems^14,37^. In these systems, the virally-encoded version of the immunity repressor contains a C-terminal *ssrA*-like tag that promotes degradation and renders it non-functional. The *attP* site is located within the repressor gene, near the 3’ end, such that integration results in a functional prophage-encoded repressor lacking the *ssrA*-like tag. Similarly, Bb423phi2 and 689b-1 both have an integrase gene adjacent to *attL* and an immunity repressor gene adjacent to *attR*; *in silico* reconstruction of the viral genomes produces viral versions of the repressor genes coding for proteins with 18 C-terminal residues that are absent from the prophage-encoded forms and which include *ssrA*-like tags (Fig. 3c)^37,38^. Bb423phi2 and 689b-1 thus appear to have intact immunity and integration functions, and may indeed be fully functioning prophages.

If prophages are competent to form infectious particles and thus move between different bacterial hosts over short evolutionary timescales, then phylogenetic congruency with their hosts is not expected. In contrast, if prophages are cryptic and inactive, they will strictly be inherited together with other host genes, to which they are phylogenetically congruent. In comparison to bifidobacterial 16S rRNA sequence similarity, at least some of the observed phage genetic differences are likely to be the result of independent integration events (Fig. 2c). For instance, *B. breve* 180W8-3, *B. breve* 139W4-23, and *B. breve* 017W4-39 are closely related hosts and yet, although Bb83phi1 and Bb439phi1 are nearly identical (and thus may reflect a single integration event), Bb423phi1 is more distantly related. Similarly, *B. longum* 157F and *B. longum* ATCC 15697 hosts are in the same clade, but Bl157phi1 is not as similar to Binf-1 as to other prophages. Additionally, Bl30698phi1 is very similar to Bb423phi1 and Bl157phi1, but its host *B. longum* CCUG 30698 is distantly related to all other *B. breve* and *B. longum* genomes. Therefore, at least some of the observed prophage genetic diversity is non-synchronous with the host diversity and is thus likely to be due to distinct integration events.

Comparative analyses suggest that one of the prophages integrated at the *dnaJ*_*2*_ site (Bl157phi1) has several nucleotide sequence variations relative to its nearest relatives, Bl30698phi1 and Bb423phi1, that potentially affect its ability to generate infectious particles. Two of these are 1,399 bp insertions of IS30-family transposons^39^: one within the virion structure and assembly operon, and one within the putative DNA replication genes (Fig. 1a). Each has 50 bp inverted repeats flanked by 3 bp direct repeats, and encodes a putative transposase (*BLIF_1064* and *BLIF_1048*, respectively). The two transposons are nearly identical, and several additional copies are present elsewhere in the *B. longum* 157F genome, suggesting that these are active and mobile. Neither insertion interrupts coding sequences, although they could have polar effects on downstream gene expression. A third variation is the absence of three genes adjacent to the lysis cassette, relative to Bb423phi1 and Bl30698phi1 (Fig. 1a). The genes are of unknown function and are flanked by a 30 bp repeated sequence, and their role in lytic growth is unclear.

### Prophage induction and genome excision

Overall, the eleven *B. breve* and *B. longum* prophages are genetically diverse, and the observed genomic characteristics suggest that they may still produce infectious particles. It is common for lysogenic cultures to spontaneously release infectious particles during growth. Therefore, cultures of *B. breve* 082W4-8, *B. breve* 180W8-3, *B. breve* 139W4–23, *B. breve* 017W4–39, and *B. breve* 215W4–47a were tested for the presence of infectious particles using plaque assays. Filtered culture supernatants from each potential lysogen were spotted onto confluent lawns of each potential lysogen, two known lysogens (*B. choerinum* LMG 10510 and *B. moukalabense* DSM 27321), and three non-lysogens [*B. breve* UCC2003; *B. breve* JCM 7017; and *B. breve* NCIMB 702258 (formerly NCFB 2258)] (see Supplementary Materials and Methods). However, similar to previous reports with *B. choerinum* LMG 10510 and *B. moukalabense* DSM 27321^19^, no plaques were observed. The absence of plaques could be due to many factors related to the phage, host, or experimental setup. Therefore, these strains were further investigated to determine the extent to which the prophages could be chemically induced and produce fully-assembled particles.

**Figure 4:**
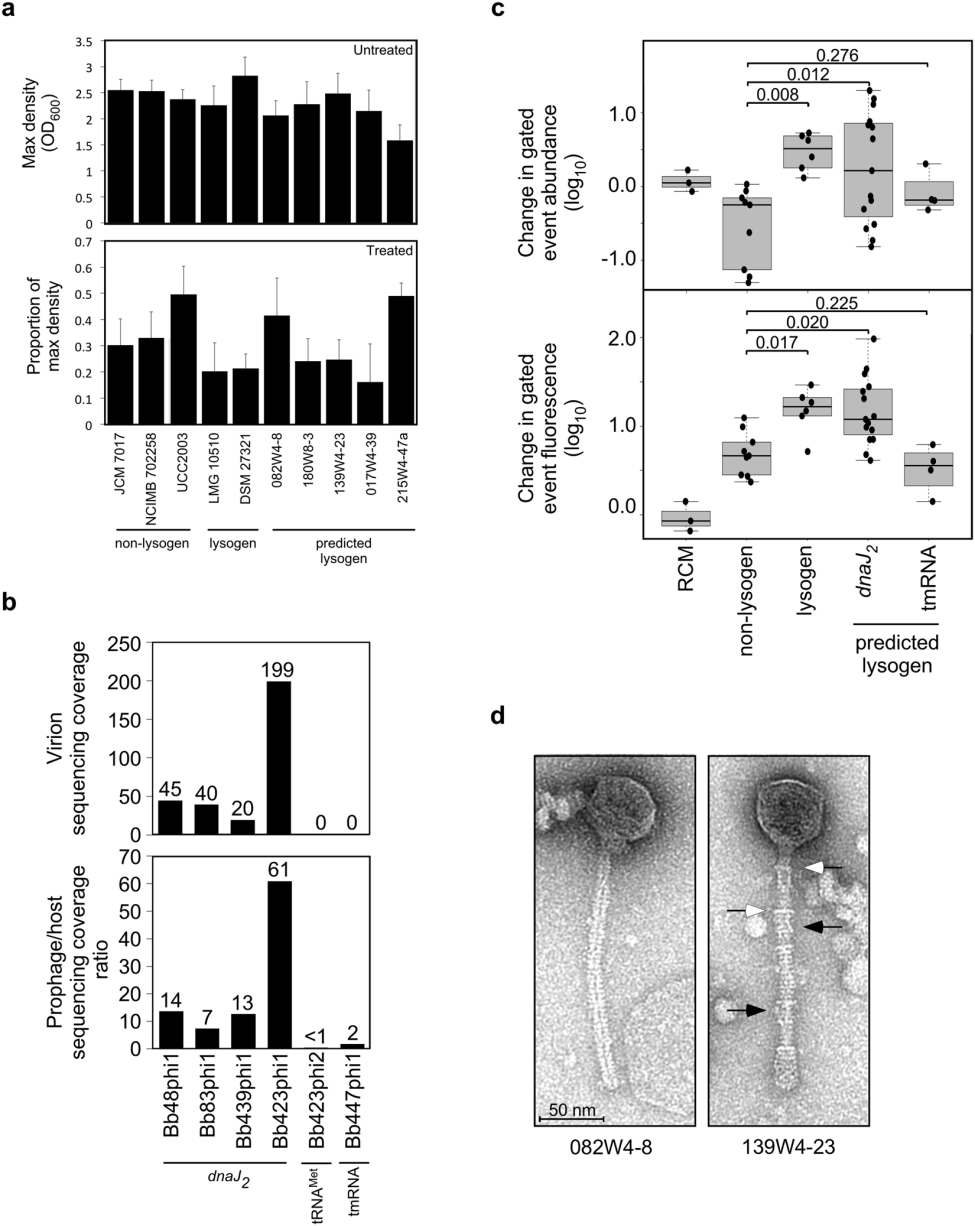
Mitomycin C induction of *dnaJ*_*2*_-integrated bifidoprophages. (**a**) Growth characteristics for several non-lysogens, lysogens, and predicted lysogens that were (top) untreated or (bottom) treated with mitomycin C. (top) Barplot displays the average maximum saturated culture density (based on OD_600nm_). (bottom) Barplot displays the effect of mitomycin C treatment on maximum culture density. Cultures were treated with 0.3 µg/ml mitomycin C at OD_600nm_ ~0.15–0.25 and after overnight incubation the final density was measured. An untreated sample was grown overnight as well, and the ratio of the treated versus untreated maximum saturated culture density was determined. Error bars indicate standard deviation from three or more replicates. (**b**) For several predicted lysogens, DNA was extracted from mitomycin C-treated culture supernatants and sequenced. Sequencing reads were used to (bottom) determine enrichment of the prophage genome relative to the host genome, and (top) assemble virion genomes and compute coverage depth. (**c**) Flow cytometry was used to quantify changes in composition of the culture supernatant after mitomycin C treatment. Replicate sets of paired treated and untreated samples for each strain were analyzed by flow cytometry (Supplementary Figs S3–5). Boxplots display fold changes in the abundance (top) and median fluorescence (bottom) of events observed from mitomycin C treated versus untreated paired samples for growth medium (RCM) and non-lysogenic, lysogenic, and predicted lysogenic strains (from Supplementary Fig. S5). Prophage integration loci in predicted lysogens are indicated. Black bar indicates median, and individual data points are plotted. Statistical significance of samples from different types of strains (lysogens, n = 6; *dnaJ*_*2*_, n = 15; tmRNA, n = 4) compared to non-lysogens (n = 9) are indicated (p-value from two-tailed t-test). (**d**) Complete phage particles identified by transmission electron microscopy in mitomycin C-treated supernatants of *B. breve* 082W4–8 and *B. breve* 139W4–23 cultures. The phage induced from *B. breve* 139W4–23 contains tail decoration discs (open arrows) and tail decoration fibers (closed arrows).

Excision and lytic induction of many prophages, including those in *B. choerinum* LMG 10510 and *B. moukalabense* DSM 27321, can be induced with mitomycin C^40^. Therefore, we tested the impact of mitomycin C on several *B. breve* strains. After initial calibration (see Supplementary Materials and Methods), mitomycin C treatment revealed a narrow window of concentrations that promotes induction of known prophages without completely inhibiting growth of non-lysogens (Fig. 4a). Addition of 0.3 µg/ml mitomycin C (similar to that used for *B. choerinum* LMG 10510 and *B. moukalabense* DSM 27321^19^) inhibited growth of all non-lysogenic strains, but had a somewhat stronger impact on the two known lysogens. *B. breve* 180W8-3, *B. breve* 139W4–23, and *B. breve* 017W4–39 strains, all of which harbor *dnaJ*_*2*_-integrated prophages, have inhibition levels similar to the known lysogens, consistent with prophage-induction in these strains. In contrast, mitomycin C addition had little impact on *B. breve* 082W4–8 and *B. breve* 215W4–47a, although we note that *B. breve* 215W4–47a grows poorly even in the absence of mitomycin C (Fig. 4a).

Successful induction is expected to result in prophage excision and circularization at the attachment junctions. Mitomycin C-induced prophage excision was assessed by PCR amplification across the predicted *attP* locus (Supplementary Fig. S1). We observed mitomycin C-dependent *attP* formation for nearly all predicted prophages, and some circularization of both Bb48phi1 and Bb423phi1 was seen even in the absence of mitomycin C, suggesting spontaneous prophage induction (Supplementary Fig. S1). These results suggest that for most *B. breve* prophages tested, the gene regulatory mechanisms controlling lysogeny are properly functioning and responsive to DNA damage signaling, although with varying induction strengths.

### Induced prophages replicate, lyse, and assemble complete particles

Following induction and excision, the phage genome is expected to replicate, thereby increasing its copy number relative to the host genome. To measure replication, DNA from culture supernatants from mitomycin C-treated cells was extracted and sequenced using Illumina MiSeq technology (see Materials and Methods). Although Bb447phi1 and Bb423phi2 exhibit a similar copy number as the host genome, *dnaJ*_*2*_-integrated prophages exhibit ~10- to 60-fold higher sequencing coverage than the host genome (Fig. 4b, Supplementary Fig. S2). The complete phage genomes induced from the *dnaJ*_*2*_ site in these four strains were assembled with 20- to 200-fold coverage (Fig. 4b). This indicates that the genome replication mechanisms are functioning in at least these *dnaJ*_*2*_-integrated prophages.

Following DNA replication, concatemers of phage genomes are typically linearized by terminase at the *cos* site during the process of virion packaging. Discontinuities in sequencing coverage across virion DNA typically correspond to genome termini resulting from this cleavage. For both Bb83phi1 and Bb423phi1, a prominent change in coverage was observed in a non-coding region upstream of the structural and assembly genes (data not shown). This position is highly conserved across most of the *dnaJ*_*2*_-integrated prophages and corresponds to the genome termini reported for phage phiSASD1 (Figs 1a, 3a). These observations suggest that for at least Bb83phi1 and Bb423phi1, and possibly other *dnaJ*_*2*_-integrated prophages, the excised and replicating genomes are packaged into virions.

Cell lysis is expected to occur after replication and packaging, increasing the quantity of DNA-containing phage particles in the supernatant. The extent of lysis and phage release can be assessed with flow cytometry. Flow cytometry has been used to identify stained, PEG precipitated *Lactococcus lactis* phages in culture supernatants after mitomycin C induction^41^. Here, a modified strategy was used to quantify mitomycin C-induced changes in supernatant composition, since phage release is expected to increase both the abundance and fluorescence of flow cytometric events (see Materials and Methods). Several non-lysogens, lysogens, and predicted lysogens were grown in RCM and treated with mitomycin C (or were left untreated) at early log phase. Culture supernatants were filtered, PEG precipitated, stained with Syto9, and processed by flow cytometry (Supplementary Figs S3–S5). When individual strains are compared, changes in abundance and fluorescence of events between treated and untreated samples do not clearly indicate phage release (Supplementary Fig. S5b). Variability is observed between replicate sets of the same strain as well as between strains of the same strain type (non-lysogen, lysogen, or predicted lysogen). This suggests that either mitomycin C treatment does not reproducibly generate distinct, robust, induction-dependent changes in supernatant composition, which is consistent with the poor and variable degree of growth inhibition observed (Fig. 4a), or it could be a result of phage aggregation during the PEG precipitation step. However, despite this variability, when results of each strain type are combined, the increases in event abundance and fluorescence for lysogens and predicted lysogens with *dnaJ*_*2*_-integrated prophages are indeed significantly larger than those for non-lysogenic strains (Fig. 4c). In contrast, *B. breve* 215W4–47a, with the tmRNA-integrated Bb447phi1, does not exhibit significant changes. Thus, as a group, lysogens harboring a *dnaJ*_*2*_-integrated prophage exhibit significant (although variable) changes in mitomycin C-dependent supernatant composition, consistent with the hypothesis that these phages are released.

Although the *dnaJ*_*2*_-integrated phages exhibit mitomycin C-dependent replication, packaging and lysis, it is not clear whether completely assembled phage particles are produced. To address this, mitomycin C-treated culture supernatants were analyzed by transmission electron microscopy (TEM) for two representative strains, *B. breve* 082W4–8 and *B. breve* 139W4–23. In culture supernatants of both strains, phage particles are indeed observed (Fig. 4d, Table 4), but they are at low concentration and at the limit of detection for TEM analysis (approx. 10^5^–10^6^ phage particles per ml). They both exhibit a morphotype consistent with (isometric-headed) *Siphoviridae* phages. For *B. breve* 082W4–8, the few phage particles detected are most likely derived from the only predicted prophage, Bb48phi1. Of the six phage particles detected, four exhibited empty capsids and two revealed intact phage heads (diameter: 60 nm) and flexible 200 nm tails. For phage particles derived from *B. breve* 139W4–23, similar head and tail dimensions were also recorded (Table 4). Notably, these phage tails were decorated with 6–7 discs (width ca. 17 nm) and with thin tail fibers attached along the whole tail surface (Fig. 4d). Even though there are two predicted prophages in *B. breve* 139W4–23, the observed phage particles are likely derived from Bb423phi1 since no evidence of excision or induction was observed for Bb423phi2 (Fig. 4, Supplementary Figs S1, S2). Thus, for these two strains, and possibly for the other lysogens with *dnaJ*_*2*_-integrated prophages, completely assembled phages can be produced. However, plaque assays using mitomycin C-induced samples again failed to generate plaques (see Supplementary Materials and Methods).

**Table 4:** Dimensions of bifidophages detected by TEM^a^.

Strain	Head (nm)	Tail length (nm)	Tail width (nm)	Tail decorations width (nm)
*B. breve* 082W4–8	60.3 ± 0.04 *(n* = *2)*	200.5 ± 2.2 *(n* = *2)*	11.8 ± 0.6 *(n* = *2)*	*Not applicable*
*B. breve* 139W4–23	60.3 ± 3.3 *(n* = *5)*	193.7 ± 1.7 *(n* = *5)*	13.4 ± 0.6 *(n* = *5)*	17.1 ± 1.0 *(n* = *8)*

^a^Due to the low number of phage particles detected, very few measurements were possible.

Only four of the *B. breve* prophages exhibited a response to mitomycin C. However, not all prophages are mitomycin C inducible, including most mycobacteriophages, such as Brujita, which use integration-dependent immunity systems similar to those in Bb423phi2 and 689b-1. These mycobacterial prophages are fully competent to form infectious particles by spontaneous phage release but are not sensitive to mitomycin C^37^. Thus, the lack of induction for some bifidoprophages does not necessarily reflect an inability to produce infectious particles.

### A putative novel shufflon conferring host range specificity

**Figure 5:**
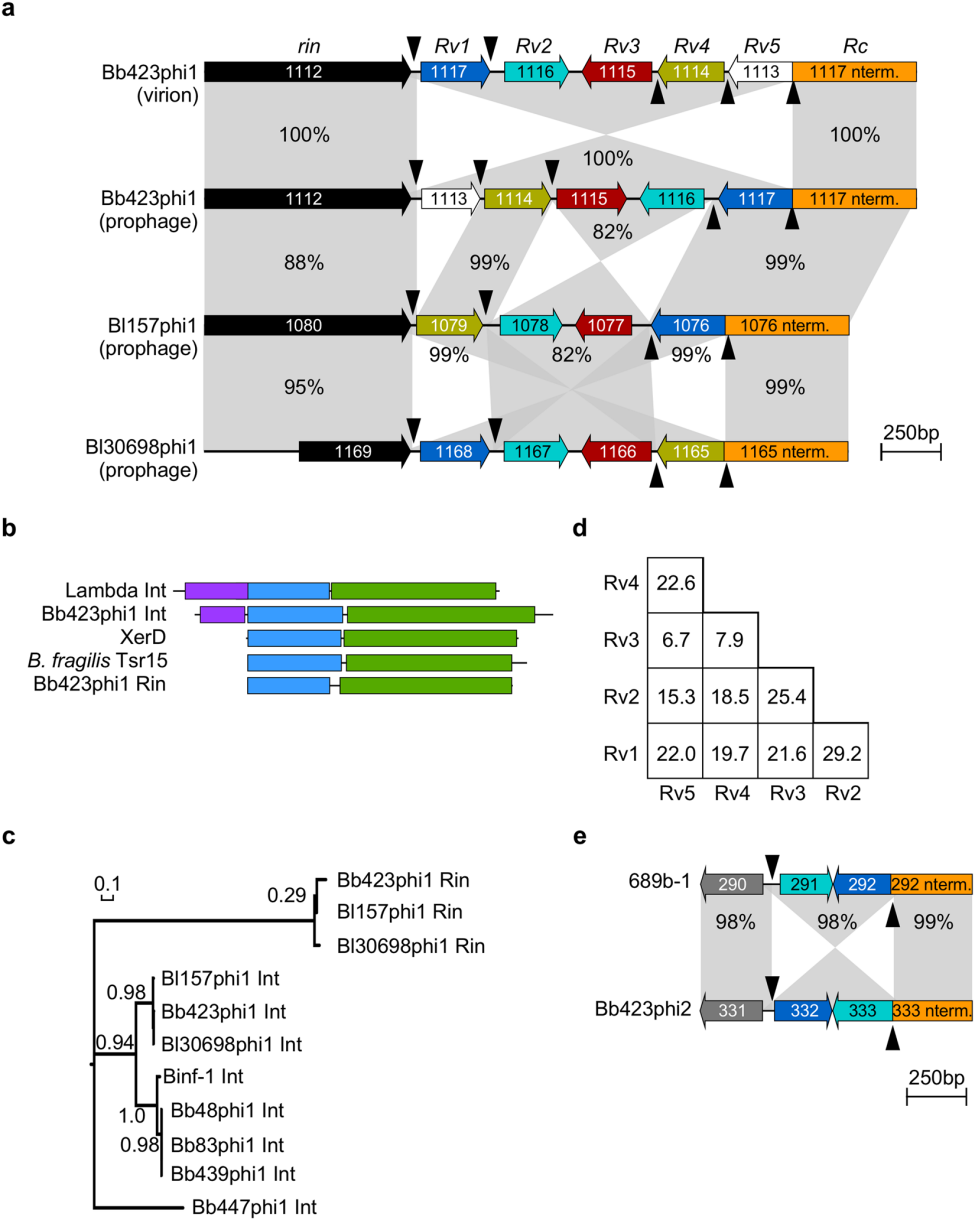
Characterization of the Rin shufflon. (**a**) Enlarged view of the Rin shufflon in several bifidophages from Supplementary Fig. S6a, with genes (arrows) oriented relative to direction of transcription and labeled with their systematic gene numbers. BLAST alignment of the Rin shufflon loci from the Bb423phi1 prophage and induced virion genomes and Bl157phi1 and Bl30698phi1 prophage genomes highlight multiple sequence inversions. Shaded regions between genomes indicate regions of homology and percent sequence identities are labeled. Rin shufflon components analogous to the Min shufflon are indicated. Variable RBP C-terminus coding regions (*Rv*) are numbered according to orientation in Bb423phi1 prophage and color-coded to highlight homologues in the Bl157phi1 and Bl30698phi1 prophages. *Rv* genes are flanked by the predicted tyrosine invertase (*rin*, black) and the constant RBP N-terminus coding sequence (*Rc*, orange). *Rv* genes are separated by putative 11 bp crossover sites (*rix*, arrowheads). (**b**) Protein domain comparison between different types of tyrosine recombinases, including Lambda integrase (int), the predicted Bb423phi1 integrase, XerD, *Bacteroides fragilis* Tsr15 invertase, and Bb423phi1 Rin. Approximate regions of the arm-type DNA-binding (purple), common core DNA-binding (blue), and catalytic (green) domains predicted by HHpred are indicated. Proteins are manually aligned by the N-terminus of the common core DNA-binding domain. (**c**) Unrooted maximum likelihood phylogenetic tree constructed from alignment of the invertases and integrases identified in eight *B. breve* and *B. longum* prophages. aLRT branch support indicated. (**d**) Matrix of pairwise Bb423phi1 Rv amino acid sequence identities. (**e**) Enlarged view from Supplementary Fig. S6b of the inverted locus in tRNA^Met^-integrated prophages, labeled as in panel (**a**).

The phage tail impacts host adsorption and specificity, and the different tail morphologies of Bb48phi1 and Bb423phi1 suggest they recognize their hosts in distinct ways. The seven *dnaJ*_*2*_-integrated prophages contain virion structural and host lysis genes in syntenic positions (Supplementary Fig. S6a). Although they have related tape measure protein (TMP)-encoding genes, there are two distinct distal tail (DIT)-encompassing gene modules. One subset (Binf-1, Bb48phi1, Bb83phi1, and Bb439phi1) contains two genes immediately downstream of the TMP gene that exhibit distant similarity to the DIT^42^ and RBP genes of *Lactococcus lactis* phage TP901-1, respectively^43^. In contrast, the other three phages (Bb423phi1, Bl30698phi1, and Bl157phi1) contain a markedly different locus, designated Rin, with features similar to the Min shufflon in the cryptic extrachromosomal coliphage p15B (Fig. 5a, Supplementary Fig. S6a)^44^. The Min system utilizes a serine-family site-specific recombinase (Min) to generate multiple DNA inversions that give rise to variable tail fiber types with different host specificities^45^. The mechanism of Min inversion is related to the simpler DNA inversion systems such as Gin (in coliphage Mu) and Cin (in coliphage P1) that also control tail fiber phase variation^46^.

The Rin loci of Bb423phi1, Bl30698phi1, and Bl157phi1 contain several small, tandemly oriented genes, most of which code for proteins with weak similarity to the *Lactococcus lactis* phage TP901-1 RBP C-terminus^43^. These RBP C-terminus variable (*Rv*) genes are flanked on one side by the RBP N-terminus constant (*Rc*) gene, which is distantly related to the RBP of *Lactococcus* phage 1358, and on the other side by a predicted recombinase, the RBP locus invertase (*rin*). Upstream of each *Rv* gene is a short, asymmetric, 11 bp repeated sequence (TTCCCTAACCC), likely encompassing the Rin crossover sites (*rix*) that facilitate inversion. These short repeats are not abundant elsewhere in the host genomes.

The putative Rin shufflon of these bifidophages differs from the Min, Cin, and Gin systems in that it utilizes a tyrosine-family recombinase rather than a serine-family recombinase. Tyrosine-specific invertases, such as Tsr15^47^, are present in *Bacteroides fragilis* genomes, which in general are replete with a variety of phase variation systems such as those that modulate restriction-modification systems^48^ or outer surface proteins^47^. The organization of the Rin recombinase itself resembles tyrosine recombinases such as XerD^49^, which contain common core-type DNA-binding and catalytic domains, but lack the N-terminal arm-type DNA-binding domain present in canonical tyrosine integrases such as of phage Lambda (Fig. 5b). The Rin recombinases are also distinct from the predicted tyrosine integrases encoded elsewhere in the prophage genomes, which have canonical Lambda Int-like organizations (Figs 1a, 5b, 5c). The absence of the arm-type DNA-binding domain in Rin raises the question as to how the directionality of recombination is regulated, such that flipping between inverted *rix* sites occurs, but deletion between directly oriented *rix* sites is avoided. This is similar to the conundrum of how recombination directionality is regulated in the armless integration-dependent phage immunity systems^50^.

The full length RBP gene contains two directly oriented *rix* sites, one within the gene itself, and a second immediately following the 3’ end of the gene (Fig. 5a). Inversion between the *rix* site within the RBP gene and any of the 2–3 *rix* sites in inverted orientation will generate a new full length RBP gene that codes for the same 168 residue N-terminus but with a different 95–100 residue C-terminus. Inversion involving the *rix* site at the 3’ end of the gene rearranges two or more *Rv* genes to prime the cassette for new full length RBP genes at subsequent inversions. Comparisons of the three prophage loci provide evidence supporting activity of these DNA inversion systems. First, Bl157phi1 contains an inversion relative to Bb423phi1, and two inversions relative to Bl30698phi1 (Fig. 5a). Second, the sequenced Bb423phi1 virion genome exhibits sequence inversions relative to the published prophage orientation (variant 1); although inversion of the entire *Rv* segment (variant 2) is the predominant arrangement, the virion DNA sequence reads reflect possibly three variant orientations (Supplementary Fig. S7). Third, the sequenced *B. breve* 139W4–23 genome^18^ also exhibits three sequence inversions (Supplementary Fig. S8). Although the majority of the DNA sequence reads reflect the published orientation (variant 1), two other variant orientations are identified, and they are distinct from those present in the induced genome sequence analysis (Supplementary Figs S7d, S8). All prophage and virion inversions occur within the identified *rix* sites (Fig. 5a), and examples of all five possible full-length RBP genes are represented in this dataset (Fig. 5a, Supplementary Figs S7d, S8). The Rv proteins are quite dissimilar from each other, ranging between ~6–30% amino acid sequence identity (Fig. 5d), as expected if the shufflon controls host range specificity.

A second putative phase variation system is present in the two tRNA^Met^-integrated phages (Fig. 5e, Supplementary Fig. S6b). Comparison of the structural genes in 689b-1 and Bb423phi2 reveals that a ~500 bp sequence in Bb423phi2 containing a small gene, *BB139W423_0332*, and the 3’-end of the adjacent gene, *BB139W423_0333*, have become inverted relative to 689b-1 (Fig. 5e). Neither gene has an identifiable function based on homology searches. Similar to the Rin system, the observed inversion occurs at an 8 bp sequence, CAGGGTTA, and the two gene segments are quite dissimilar. However, unlike Rin, no recombinase flanks the locus. Some invertases, such as the *Bacteroides fragilis* Mpi serine invertase, can act globally to facilitate inversions in *trans*^51^. Thus, this second bifidophage inverted locus may be a simpler phase variation system that relies on a DNA invertase supplied in *trans*, which has not been previously reported in phage genomes.

Phase variation systems enhance the diversity of phage tail structural proteins to promote rapid switching of bacterial hosts^44,46^. The two putative bifidophage inversion systems represent the first such systems identified in phages infecting actinobacterial hosts and may exhibit unique properties. They may also help to explain why bifidophage plaques are difficult to observe. Bacterial hosts harbor numerous phage defense strategies, including CRISPR-Cas^23^, restriction modification systems^18^, and prophage-encoded systems^52^. It is plausible that the bifidobacterial host strains prevent phage infection by actively generating variations in cell wall components that are used as phage receptors. The dynamic interplay between variability of both host and phage moieties likely contributes to the inability to find plaque-forming bifidophages^19–21^. Future studies are needed to confirm activity of these inversion systems and their role in host specificity.

### Bifidophage evolutionary history

Recently, it has been shown that there are two classes of temperate phages, marked by distinctly different rates of gene content flux (GCF)^53^. In general, the seven *dnaJ*_*2*_-integrating prophages exhibit high GCF characteristics, and several of them can be classified as Class 1 (Supplementary Fig. S9). Thus, this group of bifidophages may experience more frequent horizontal gene exchange than other Class 2 temperate phages.

**Figure 6:**
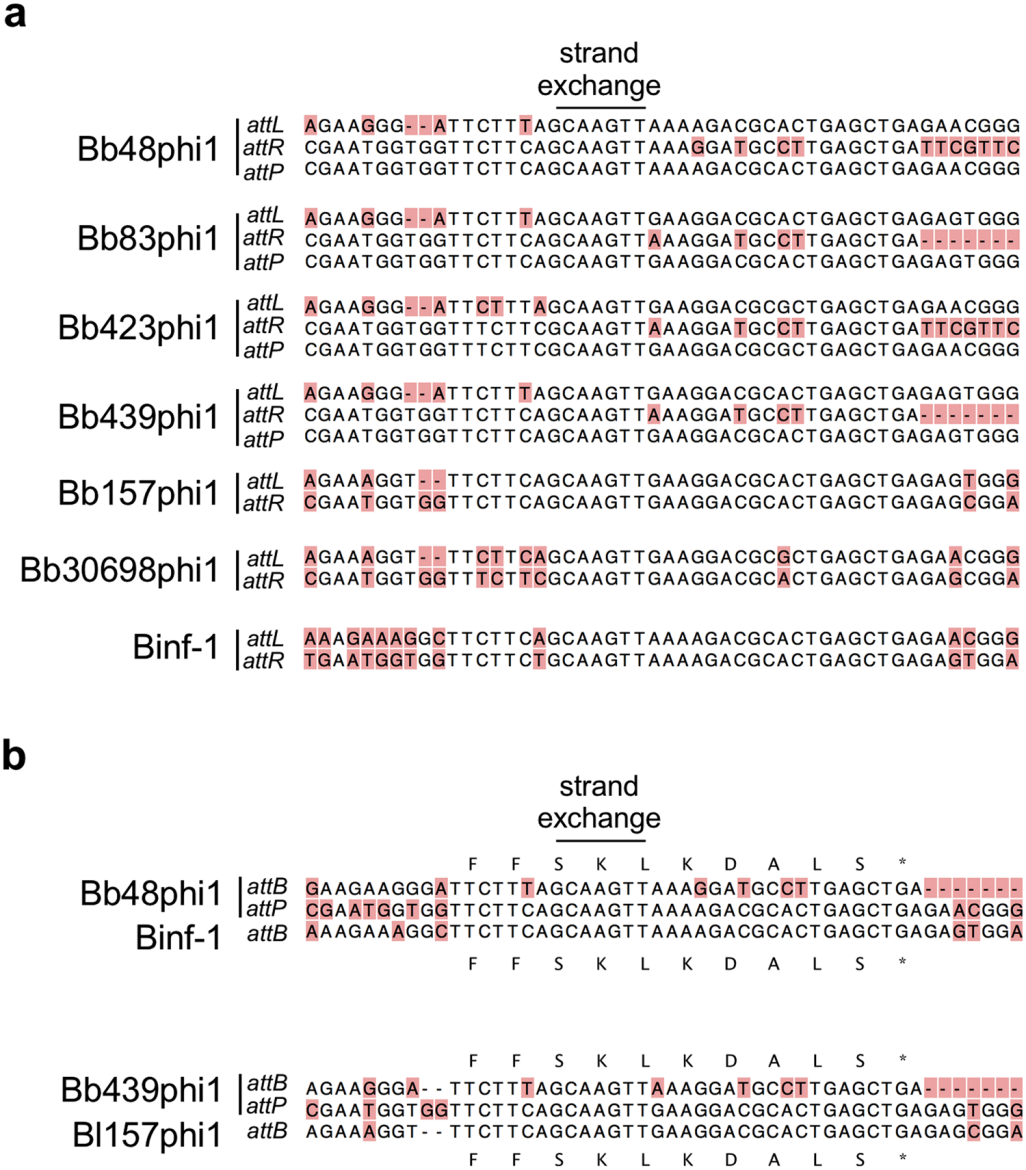
*dnaJ*_*2*_-integrated bifidoprophage attachment site analysis. **(a**) For each *dnaJ*_*2*_-integrated phage, the *attL* and *attR* sites, as well as their *attP* sites (if they were induced and sequenced), were aligned to determine the site of strand exchange during integration and excision and to deduce the *attB* sequence. Variant nucleotide positions are highlighted (beige). (**b**) The *attP* sites of Bb48phi1 and Bb439phi1 are aligned to the *attB* sites of their originating *B. breve* hosts and *B. longum attB* sites used by phages Binf-1 and Bl157phi1.

Additionally, comparison of integration sites also highlights host range dynamics. Tyrosine integrases, such as found in mycobacteriophage L5^54^, utilize a “common core” homologous sequence present in both the *attP* and *attB* sites to facilitate integration. Although only 7–8 bp of homology between the attachment sites is required for strand exchange to facilitate integration and excision, it is common for attachment sites to have more extended segments of sequence identity. This is observed for the tmRNA-integrated prophages, the tRNA^Met^-integrated prophages, and *B. longum dnaJ*_*2*_-integrated prophages, which have common cores of 26 bp, 39 bp, and 35 bp, respectively, and that exhibit only 0–2 mismatches (Table 3). However, most of the *dnaJ*_*2*_-integrated prophages have 5–7 mismatches across the 35 bp common cores at their attachment sites (Fig. 6a). To investigate this, the four virion *attP* sites were aligned with their respective *attL* and *attR* sites (Fig. 6a). There is a 7 bp region completely conserved in these sites as well as in the *attL* and *attR* sites of the other three *dnaJ*_*2*_-integrated prophages, which likely encompasses the points of strand exchange. Using this crossover point, the putative *attB* sites for all seven phages, and the predicted *attP* sites for all untested phages, were generated and compared. The *attP* sites of the four *B. breve dnaJ*_*2*_-integrated phages, such as Bb48phi1 and Bb439phi1, are more similar to the *B. longum attB* sites than the *B. breve attB* sites of their parent strains (Fig. 6b). This suggests that the four *B. breve dnaJ*_*2*_-integrating phages are in close genetic contact with hosts of both species. They may be able to integrate into either species, since integration in these strains results in reconstruction of a functional *dnaJ*_*2*_ protein. The extended identity between the phage and *B. longum* sequences could have arisen from a recent gene conversion event in a *B. longum* lysogen.

## Conclusions

Bifidobacteria play important roles in the human gut microbiome, and their bacteriophages are expected to influence their microbial communities. We have characterized eleven *B. breve* and *B. longum* prophages integrated at three chromosomal loci, and although we have not been able to show here that any of them do indeed form infectious particles, the bioinformatic analysis, mitomycin C induction profiles, transmission electron microscopy, and the presence of genetically active systems for switching host preferences, are all consistent with this interpretation. The diversity of the bifidobacterial prophages characterized here will facilitate our understanding of the gut microbiome and contribute to the interpretation of related sequences in metagenomic and metaviromic studies. They represent excellent starting points to systematically search for susceptible hosts as well as to develop tools to advance bifidobacterial genetics.

## Materials and Methods

### Bacterial strains

Strains used in this study are described in Table 1.

### Prophage characterization

Prophages present in *B. breve* 082W4-8, *B. breve* 180W8-3, *B. breve* 139W4-23, *B. breve* 017W4-39, and *B. breve* 215W4-47a strains were previously reported^18^. Prophages integrated at the homologous locus in other *B. breve* and *B. longum* strains were identified by BLAST^26^ using the predicted integrases. Gene functions were predicted with BLAST^26^ and HHpred^27^. ProgressiveMauve^55^ whole genome alignment was used to identify integration sites, prophage sizes, and attachment sites. Prophage genomes were extracted from the host genome, and their nucleotide sequence and gene content were compared using Gepard^56^ dotplot analysis and Phamerator. The phylogenetic analysis using whole genome alignments to compare newly identified bifidoprophages with previously reported bifidoprophages was performed as previously described^19^.

### Phamerator database construction

The database Actinobacteriophage_1060 was created using Phamerator^28^, consisting of 1,060 actinobacteriophages and prophages, and is available online (http://phamerator.webfactional.com/databases_Hatfull). Genes are grouped into related gene phamilies (“phams”) using kclust^57^.

### Growth and mitomycin C induction

Bifidobacterial strains were grown in 10 ml Reinforced Clostridial Medium (RCM) in a conical tube inoculated directly from freezer stock and grown to saturation overnight at 37 °C in an anaerobic chamber. For mitomycin C induction tests, 50 ml RCM was inoculated from saturated culture at an OD_600nm_ ~ 0.05, inverted several times for gentle mixing, and grown at 37 °C in anaerobic chamber for 4–5 h without shaking. When the culture reached an OD_600nm_ of 0.15–0.25, mitomycin C was added to 0.3 μg/ml, inverted several times for gentle mixing, and incubated at 37 °C in an anaerobic chamber for 15–20 h. Final OD_600nm_ was recorded and the entire culture was centrifuged in a table-top centrifuge with swinging bucket rotor at 9,148 × *g* for 20 min with slow deceleration. Supernatant was transferred to a 50 ml syringe, filtered using a 0.45 μm filter, and stored at 4 °C. Each sample was paired with an untreated control in which the 50 ml culture was allowed to grow to saturation in the absence of mitomycin C.

### Induced phage genome sequencing

DNA from 2 ml filtered culture supernatant (described above) was extracted for sequencing by incubating with 4 μl DNase I at room temperature for 1 h, then proceeding with the Norgen Phage DNA Extraction Kit according to manufacturer’s protocol. DNA was sequenced using Illumina MiSeq technology (GenProbio, Parma, Italy), and the MEGAnnotator pipeline was used for *de novo* assembly^58^. Other than the phage genomes, the only other assembled DNA molecule observed was a 6.5 kb plasmid in the *B. breve* 082W4–8 sample. For Rin shufflon variant analysis, sequencing data were analyzed with Newbler assembler and Consed 454ContigGraph output.

### Induced phage replication quantification

Sequencing reads were trimmed at both ends with Cutadapt (https://cutadapt.readthedocs.org) using the quality score option and a value of 30. Trimmed reads were mapped with Bowtie2^59^, all non-unique reads were discarded using sed, and the data was processed with SAMtools^60^ and BEDtools^61^. Reads were mapped to the published lysogen sequence and visualized with IGV^62^. To quantify enrichment of the induced phage relative to the host, average coverage per genome was computed by dividing the number of base pairs mapped by the total size of the host or prophage genome, and fold increase in coverage was computed by dividing the average coverage of the prophage genome by the average coverage of the host genome.

### Transmission electron microscopy

Negative staining of phage particles in filtered, mitomycin C-treated, culture supernatants (described above) was performed on freshly prepared ultra-thin carbon films with 2% (w/v) uranyl acetate as previously described^63^. Micrographs were taken using a Tecnai 10 transmission electron microscope (FEI Thermo Fisher, Eindhoven, The Netherlands) at an acceleration voltage of 80 kV with a MegaView G2 CCD-camera (emsis, Muenster, Germany).

### Flow cytometry sample preparation and processing

Sample preparation and processing for flow cytometric analysis was performed similarly to previously described methods^41^. Strains were grown in RCM to early log phase, treated with mitomycin C (or were left untreated), and filtered (described above). Paired treated and untreated samples were processed in parallel. 25 ml of filtered supernatant of treated/untreated cultures were incubated with 2.5 g PEG8000 on a shaker overnight at 4 °C and spun in a Sorval centrifuge at 17,620 × *g* for 15 min at 4 °C. The supernatant was discarded, and the pellets were resuspended with 1 ml TBT buffer and transferred to a 1.5 ml tube. Samples were processed by spinning in a microcentrifuge at 10,000 × *g* for 4 min, washed twice with 1 ml ¼ strength Ringer’s solution, incubated at room temperature for 30–60 min, washed once more, and resuspended in 1 ml ¼ strength Ringer’s solution. Pellets ranged in size and opacity across strains, and they were diluted 1:10 or 1:100 with ¼ strength Ringer’s solution as needed for FACSCalibur flow cytometry. Using the Live/Dead BacLight Kit (Thermo Fisher), 100 μl of sample was diluted with 888.5 μl ¼ strength Ringer’s solution, incubated at room temperature in the dark for 15 min with 1.5 μl Syto9 dye, and spiked with 10 μl microsphere bead standards. Samples were processed with a FACSCalibur. Forward scatter (FSC-H), side scatter (SSC-H), and fluorescence (FL1-H) parameters were measured using instrument settings that were calibrated to reproduce mitomycin C-treated *Lactococcus lactis* UC509.9 and NZ9000(TP901-1) induction results reported previously using a different flow cytometer (Supplementary Fig. S3a)^41^. Several types of controls were used for downstream analysis, including distilled H_2_O, ¼ strength Ringer’s solution, and ¼ strength Ringer’s solution with beads, with and without mitomycin C added. For each sample, 100,000 events were analyzed at a rate of ~3,500–5,000 events/second. All strains were grown in RCM for direct comparison, although this medium produces higher flow cytometry background than other growth media (Supplementary Fig. S3a–c).

### Flow cytometry data analysis

FACSCalibur data were analyzed with R (version 3.4.2) (http://www.R-project.org) using Rstudio (version 1.0.153) (http://www.rstudio.com) and the flowCore^64^ and flowWorkspace^65^ packages. Flow cytometric analyses of different phage types have shown that since the standard 488 nm wavelength is larger than the average phage particle, forward and side scatter parameters do not correlate with phage size^66^. Also, fluorescence intensity of stained particles does not correlate with genome size^66^. Therefore, flow cytometry events due to debris or bead standards were gated and removed similarly to previously described methods^41^. To define the gates, the signal distribution of each parameter (FSC-H, SSC-H, FL1-H) was analyzed in several control samples to identify the signal range associated with each event type (Supplementary Fig. S3d). This resulted in debris event boundaries of FSC-H (-Inf, 50), SSC-H (-Inf, 100), and FL1-H (-Inf, 15) and bead event boundaries of FSC-H (150,1000), SSC-H (800,2700), and FL1-H (15,90). Three-dimensional gates using these boundaries account for nearly all debris or bead events in the control samples. For all test samples, events passing through either the debris or the bead gates are removed, and the remaining “gated” events are used for downstream analysis of phage induction (Supplementary Figs S3e, S4). For each paired (treated/untreated) sample, the fluorescence intensity of gated events and the ratio of gated events to total events were quantified (Supplementary Fig. S5a). To assess patterns of induction, changes in the gated/total event ratio and the median fluorescence were computed using replicate data either for each strain or for each strain type (Supplementary Figs S5b, Fig. 4c). Statistical significance was computed with the two-tailed t-test function in R.

### Rin shufflon analysis

Tyrosine recombinases were analyzed with HHpred using the PDB_mmCIF70 database, and representative domain hits were chosen to illustrate the approximate domain boundaries (N-terminal arm-type DNA-binding = 3JU0_A, 3JTZ_A; common core DNA-binding = 2OXO_A, 3NRW_A, 3LYS_B; catalytic = 5DOR_A, 1AE9_A, 5DCF_A). A phylogenetic tree of recombinases was constructed using PhyML from a codon alignment by webPRANK^67^. A stop codon is present in the middle of the Bl30698phi1 *rin* gene due to a point mutation or sequencing error. For phylogenetic purposes, the point mutation was changed to match the other alleles and the full-length gene was analyzed. For *Rv* analysis, the nucleotide sequences of all potential full-length *Rc-Rv* alleles were created, in which *Rc* was fused to each separate *Rv* sequence using the identified upstream *rix* site as the point of fusion. Full length translations were analyzed with HHpred using the PDB_mmCIF70 database. The N-terminus Rc region exhibits similarity to the RBP of *Lactococcus lactis* phage 1358 (domain hit 4L9B_A). The C-termini of all Rc-Rv protein fusions, except for Rc-Rv5, exhibit similarity to the RBP of *Lactococcus lactis* phage TP901-1 (domain hits 4IOS_A, 4HEM_C, 2F0C_A). Pairwise amino acid sequence similarities of the variable C-terminus were computed using the EMBOSS Needle global alignment tool^68^.

### *dnaJ*_*2*_-integrating phage attachment site analysis

For the induced *dnaJ*_*2*_-integrated prophages, the 7 bp point of strand exchange was determined by aligning the *attL* and *attR* sites with the *attP* site from the induced virion genome. Using the point of strand exchange, the theoretical *attB* and *attP* sites in *B. breve* and *B. longum* host strains and virion genomes were created for all other *dnaJ*_*2*_-integrated prophages.

### Host 16S rRNA analysis

The annotated 16S rRNA genes from bifidobacterial genomes were aligned with MUSCLE. The alignment was trimmed at both ends using CLC Genomics (www.clcbio.com), and a phylogenetic tree was constructed using the BioNJ algorithm in Seaview^69^.

## Electronic supplementary material

Supplementary Information

## Data Availability

The raw flow cytometry data files and the R code used for flow cytometry analysis are available upon request.
