# Supplementary material for: Characterization and induction of prophages in human gut-associated Bifidobacterium hosts
Source: Sci Rep. 2018 Aug 24;8:12772. doi: 10.1038/s41598-018-31181-3 (PMC6109161; doi:10.1038/s41598-018-31181-3)
Supplement: Supplementary file 1 — Supplementary Information [file 41598_2018_31181_MOESM1_ESM.pdf]

Characterization and induction of prophages in human gut-associated *Bifidobacterium* hosts

Travis N. Mavrich, Eoghan Casey, Joana Oliveira, Francesca Bottacini, Kieran James, Charles M.A.P. Franz, Gabriele A. Lugli, Horst Neve, Marco Ventura, Graham F. Hatfull, Jennifer Mahony, Douwe van Sinderen

Supplementary Information

Supplementary Materials and Methods

Optimization of mitomycin C induction. Mitomycin C concentration was optimized using 96-well microtiter plates. Wells with 500 μl RCM were inoculated from *B. breve* JCM 7017, *B. breve* UCC2003, and *B. breve* 017W4-39 cultures, grown for 8 h, and treated with a 10-fold serial titration of mitomycin C (ranging from 0.0003 μg/ml to 3 μg/ml) for 14 h. Growth inhibition was observed for concentrations at and above 0.03 μg/ml. Similar inhibitory profiles were observed with 0.03 μg/ml mitomycin C for 2.5 ml, but not 50 ml, cultures. Therefore, for 50 ml cultures, 0.3 μg/ml mitomycin C was used.

Induction verification using PCR. Prophage induction was confirmed by PCR amplification across the *attP* site that forms after excision and circularization. One pair of primers was designed to test *dnaJ*$_2$-integrated phages (5'-TCCGTAAAAACAGGTTAAAAACCG-3', 5'-AAACGTTGGAATCACGCCATTCC-3'). Separate primer pairs were designed for Bb447phi1 (5'-GTCACACCCACCAGAATCATGAATC-3',5'-GTTAAGAAGACTTGCTGATGGAGTTG-3') and Bb423phi2 (5'-CGAACCACTGTGTCATCATCTC-3', 5'-AGCGAGATAACTTGGACGATCAAC-3'). Induction of previously described prophages in strains *B. choerinum* LMG 10510 and *B. moukalabense* DSM 27321 was confirmed using previously described primers[19]. Amplification proceeded in 25 μl reactions containing 1 μl filtered supernatant with Taq polymerase according to manufacturer's instructions, using a thermocycler protocol of 25-30 cycles of denaturation at 94°C for 30 s, annealing at 55°C for 30 s, and extension at 72°C for 1 min.

Plaque assays. Plaque generation was attempted using a variety of phage samples, indicator strains, and growth media. To test for spontaneous phage release, filtered supernatants of saturated cultures were used. To test for mitomycin C-induced phage release, filtered supernatants or PEG precipitated samples from mitomycin C-treated cultures (as generated for

flow cytometry) were used. Confluent lawns were prepared by mixing 4.5 ml Reinforced Clostridial Top Agar (30 ml Reinforced Clostridial Agar + 60 ml RCM, with or without 2 mM $CaCl_2$) with 200-300 μl saturated bifidobacterial culture (grown overnight directly from a freezer stock) and allowed to solidify on RCA plates. For each phage sample, 3-5 μl was spotted onto the overlay and allowed to dry, and plates were incubated at 37°C in an anaerobic chamber for 24-48 h. Phage samples were generated from several lysogens (*B. choerinum* LMG 10510 and *B. moukalabense* DSM 27321) and predicted lysogens (*B. breve* 082W4-8, *B. breve* 180W8-3, *B. breve* 139W4-23, *B. breve* 017W4-39, and *B. breve* 215W4-47a), and they were tested against all of the originating lysogens and predicted lysogens as well as several non-lysogens (*B. breve* JCM 7017, *B. breve* NCIMB 702258, and *B. breve* UCC2003). As an alternative to spotting, some saturated cultures were directly mixed with phage samples in a 1.5 ml tube and aerobically incubated on the bench at room temperature for 10-15 min prior to being mixed with top agar and poured as an overlay. Additionally, TOS media (Sigma) was used as an alternative to RCM.

Rin shufflon inversion analysis in uninduced Bb423phi1. Genomic inversions within the Rin shufflon of the uninduced Bb423phi1 prophage were identified in previously reported *B. breve* 139W4-23 raw whole genome sequencing reads[18]. Pacbio long reads (average read length >10 kb) that map across the Rin shufflon locus (coordinates 1,307,900 to 1,310,888) with at least 80% sequence identity were selected using BLAT aligner v36x2. Variant shufflon orientations in this subset of reads were identified using dotplot alignments in mummer v3.0. Sequence coverage of each variant was computed using the identified long reads as reference sequences and performing a mapping assembly using the RS_Resequencing.1 protocol implemented in SMRT Analysis portal v2.3. The resulting assembled reads were inspected using the Next Generation Sequencing (NGS) visualization tool Tablet (https://ics.hutton.ac.uk/).

Gene content flux analysis. Changes in gene content and nucleotide sequence similarity were computed as previously described[53]. Briefly, for each bifidophage genome, pairwise nucleotide distances to all other actinobacteriophages were computed using Mash, and gene content dissimilarities were computed using pham designations in Phamerator. Each phage's evolutionary mode is predicted by assessing the distribution of pairwise genomic similarities using previously determined mode boundaries.

Supplementary Figure Legends

Supplementary Figure S1. Mitomycin C treatment increases bifidoprophage excision. Excision and circularization of the predicted prophages were examined by PCR. **a**, Primers (arrows) were designed in all predicted prophages so that they are divergent in the integrated genome orientation but convergent in the excised and circularized genome to amplify across the *attP*. **b**, Prophage induction was tested in several strains by PCR amplification of filtered culture supernatants (F.S.) treated (+) or not treated (-) with mitomycin C. Three to four replicates were tested per strain. For each prophage of interest, a no template control (NTC) and several unfiltered saturated cultures (S.C.) were included as negative and positive controls. The full length of each lane from the loading well to leading edge is displayed. A star (*) indicates the expected band size corresponding to *attP* amplification.

Supplementary Figure S2. Mitomycin C treatment increases *dnaJ*$_2$-integrated bifidoprophage copy number. DNA from mitomycin C-treated culture supernatants was sequenced for several *B. breve* strains and reads were mapped to the lysogen genome (black line). Enlarged view of the integrated prophage (white box) locus in each strain highlights the increased sequencing coverage of the prophage relative to the host genome.

Supplementary Figure S3. Flow cytometry calibration and gating strategy. a, FACSCalibur settings were calibrated using mitomycin C-treated *L. lactis* non-lysogen (strain UC509.9) and lysogen (strain NZ9000 with TP901-1 prophage) samples. Scatterplots comparing forward scatter (FSC-H) to (bottom) side scatter (SSC-H) and (top) Syto9 fluorescence (FL1-H) were adjusted to reproduce previously described results[41]. **b**, Flow cytometry of several negative controls, plotted as in panel **a**, to identify different types of events to gate. Samples include flow sample buffer (¼ strength Ringer's solution), flow sample buffer with reference microsphere

beads (¼ strength Ringer's solution + Beads), and growth medium processed with the entire protocol (RCM). **c**, Flow cytometry of common bifidobacterial growth media (MRS, MMRS + Glucose, TOS), plotted as in panel **b**. **d**, Boxplots of individual parameters (FSC-H, SSC-H, FL1-H) from flow cytometry results for several strain-free controls are used to define boundaries of each parameter for debris (beige) and bead (blue) events. Some samples have been treated (+) or not treated (-) with mitomycin C (MmC), Syto9 stain, and beads. **e**, Boundaries defined in panel **d** were used to create three-dimensional debris and bead gates. The gating strategy for all flow cytometry samples utilizes these two gates for removal of debris events followed by removal of bead events. All non-debris and non-bead "gated" events are used for downstream analysis to assess levels of prophage induction.

Supplementary Figure S4. Flow cytometric analysis of mitomycin C-treated bifidobacterial samples. Gated events from one representative replicate of each sample type are plotted. Scatterplots of FSC-H and SSC-H (left) and density plots of FL1-H (right) comparing events either between mitomycin C-treated *L. lactis* non-lysogenic UC509.9 (blue) and lysogenic NZ9000(TP901-1) (red) strain samples or between mitomycin C-treated (red) and untreated (blue) bifidobacterial growth medium (RCM) and strain samples.

Supplementary Figure S5. Mitomycin C induced changes in supernatant composition. a, For all gated events from each replicate set of paired mitomycin C treated (red) and untreated (blue) samples (from Supplementary Fig. S4), (top) barplot of the proportion of total events and (bottom) boxplots of event fluorescence (FL1-H) highlight changes in supernatant composition. Replicate sets are numbered. Individual strain names are indicated along with whether they are non-lysogens, lysogens, or predicted lysogens. RCM = growth medium with no cell culture. *L. lactis* untreated = mitomycin C-treated non-lysogen (UC509.9); *L. lactis* treated = mitomycin C-

treated lysogen (NZ9000 with TP901-1 prophage). **b**, Boxplots display the fold changes in the (top) abundance and (bottom) median fluorescence of events from paired samples in panel **a**.

Supplementary Figure S6. Bifidoprophages contain phase variation systems. a, Enlarged view of the left arm genes of *dnaJ*$_2$-integrated prophages from Fig. 1a highlights the genomic context of the Rin shufflon. Genes are colored according to pham designation, and any putative functions are listed (TMP = tape measure protein; DIT = distal tail protein; RBP = receptor binding protein). The color spectrum between genomes is the same as in Fig. 1a. **b**, Enlarged view of the left arm genes of tRNA^{Met}-integrated phages from Fig. 1c highlights a putative phase variation system in these genomes.

Supplementary Figure S7. Bb423phi1 induced phage genomes harbor multiple Rin shufflon variants. **a**, Three contigs (numbered by size) representing the entire phage genome are assembled by Newbler, but a 100% consensus of the complete genome is not achieved. Contigs can be connected in multiple arrangements due to reads mapping across more than one contig, and these discrepant reads occur near or within the RBP locus (colors and gene numbering as in Fig. 5a). **b**, One possible contig orientation involves reads that straddle the three contigs (dashed lines) with approximately equal coverage. **c**, Other contig orientations are possible, but they are represented by much lower read coverage and they do not obviously assemble into a single alternative genome. **d**, Two sequential inversion events (double arrows) at *rix* sites result in three shufflon variants that sufficiently account for all hybrid sequence reads.

Supplementary Figure S8. Bb423phi1 uninduced prophage genomes harbor multiple Rin shufflon variants. Analysis of the previously reported *B. breve* 139W4-23 genome sequencing reads[18] reveals three variant orientations of the Bb423phi1 prophage Rin shufflon. (Left) The variant nucleotide sequence orientations were assembled and all reads in the sample were

mapped to each variant. The genome map below the histogram and the coordinates above the histogram reflect the predominant variant in the published genome. The points of inversion in each variant are indicated below (double arrows and coordinates). Average coverage across each variant is indicated by the dotted line, and the percentage of all reads in the sample that map to the variant orientation is indicated. (Right) Dotplot sequence comparison of each variant to the published prophage locus orientation highlights the points of inversion.

Supplementary Figure S9. *dnaJ$_2$*-integrated prophages exhibit high gene content flux. Pairwise comparisons (black circles) of nucleotide sequence and gene content between *dnaJ$_2$*-integrated phages and all other actinobacteriophages as previously described[53] to highlight gene content flux patterns, with high (HGCF, blue) and low (LGCF, green) gene content flux regions indicated.

a

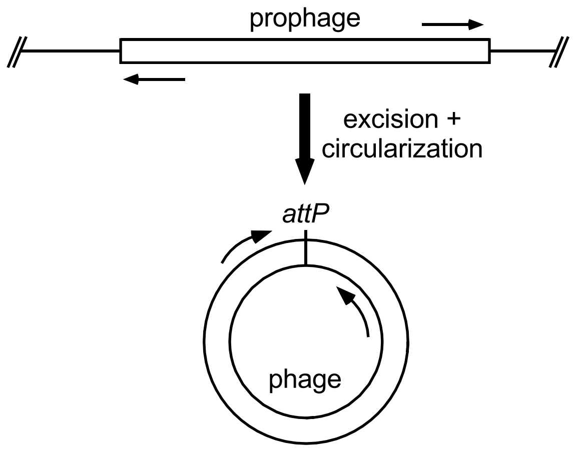

b

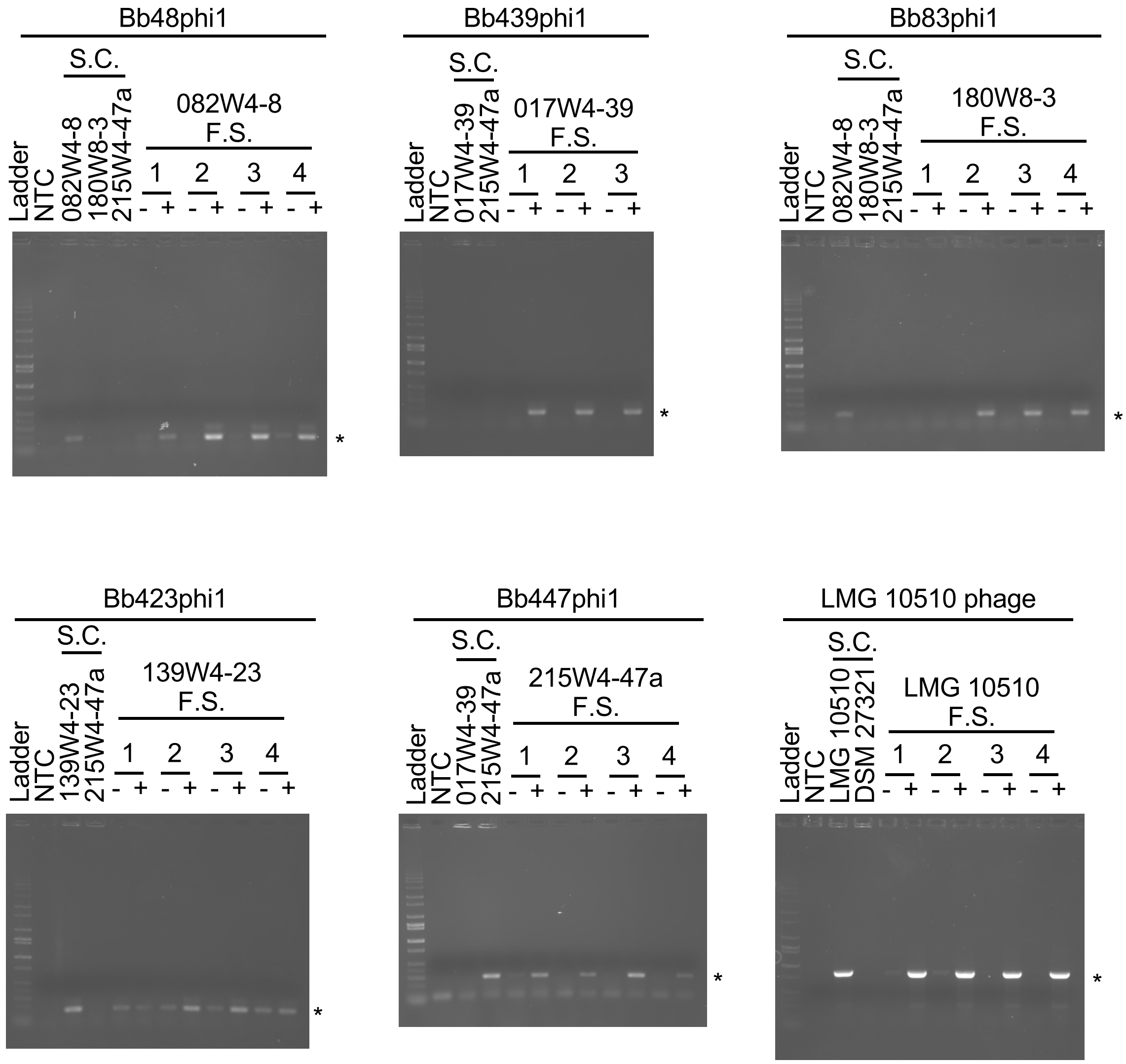

Supplementary Figure S1

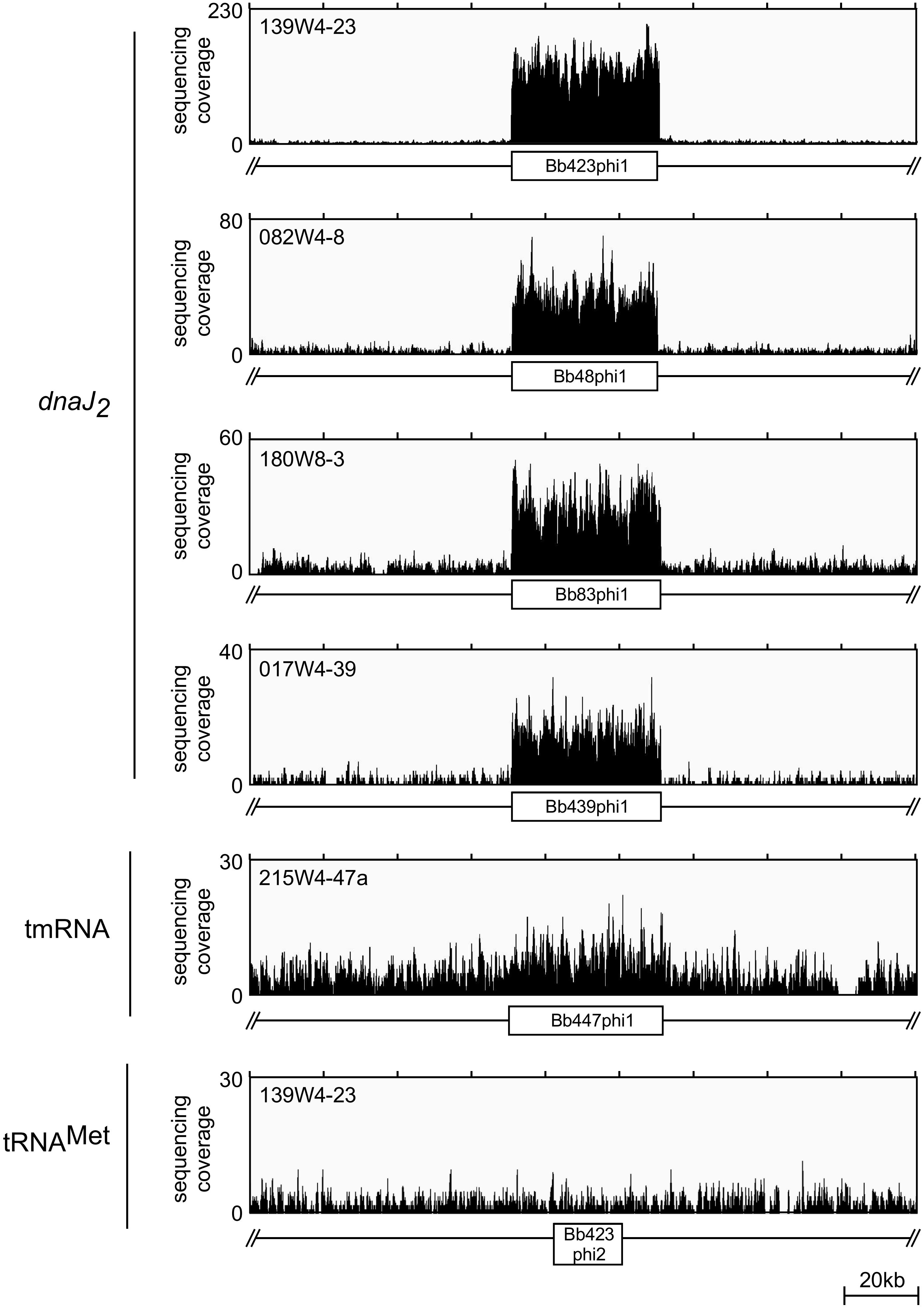

Supplementary Figure S2

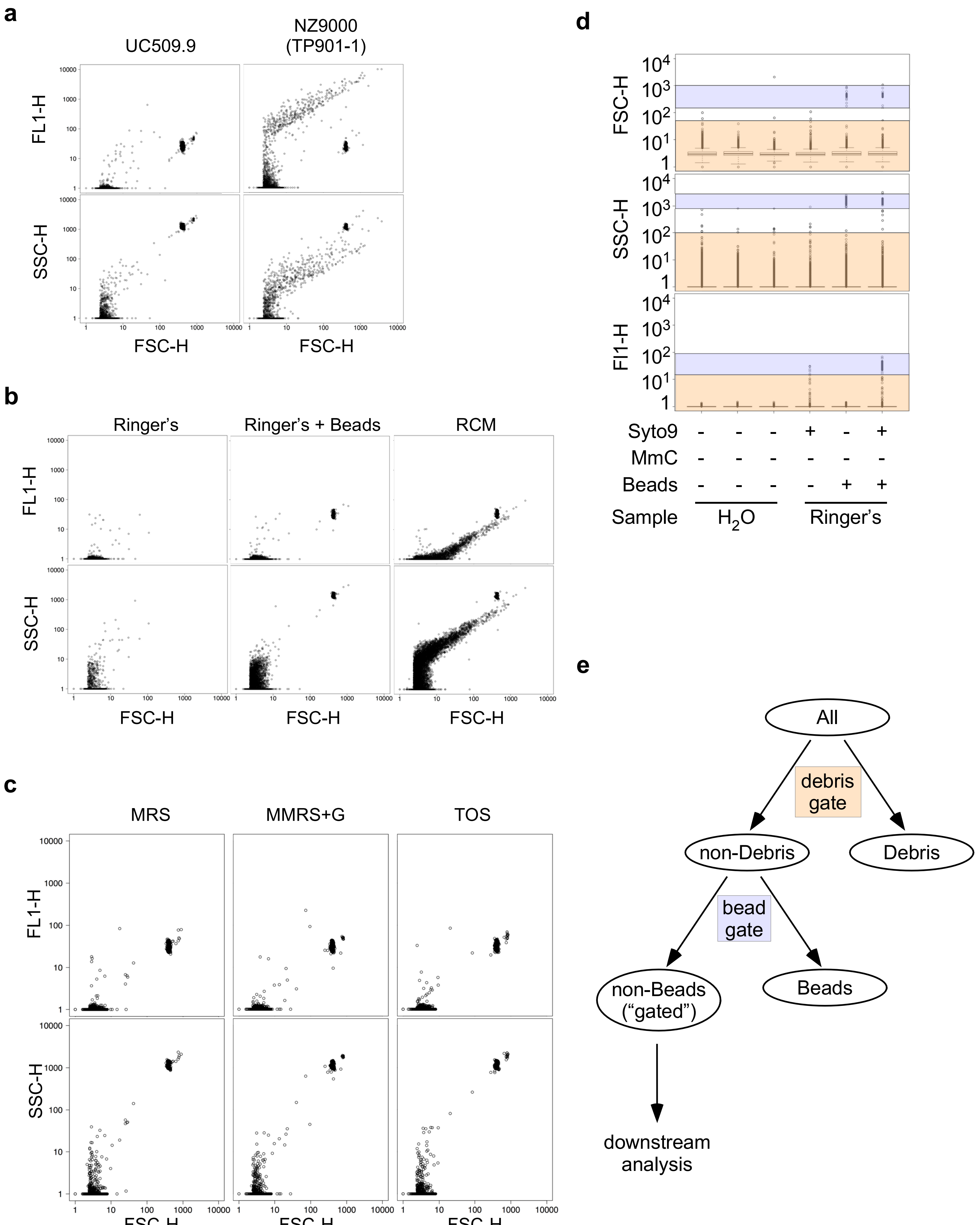

Supplementary Figure S3

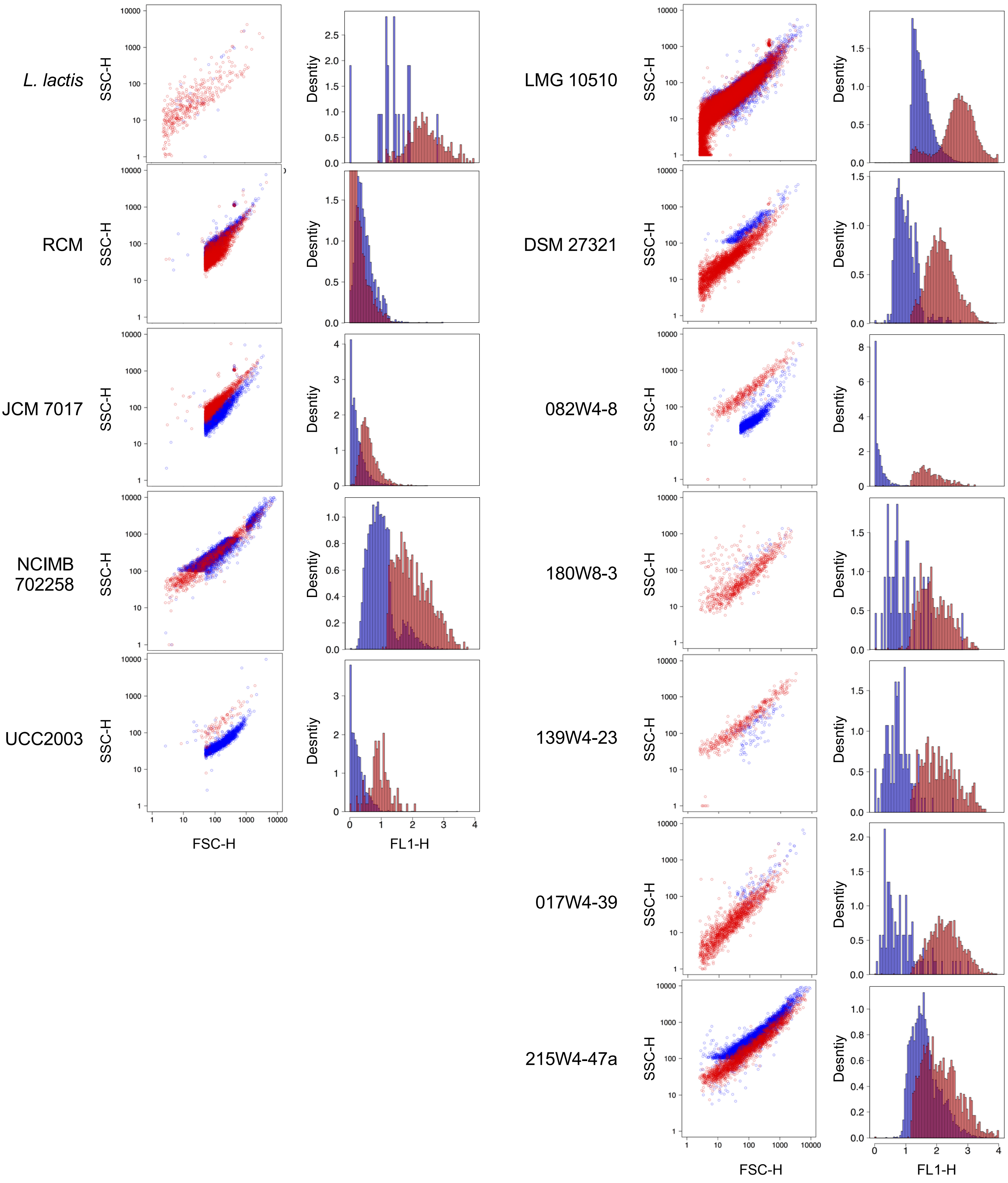

Supplementary Figure S4

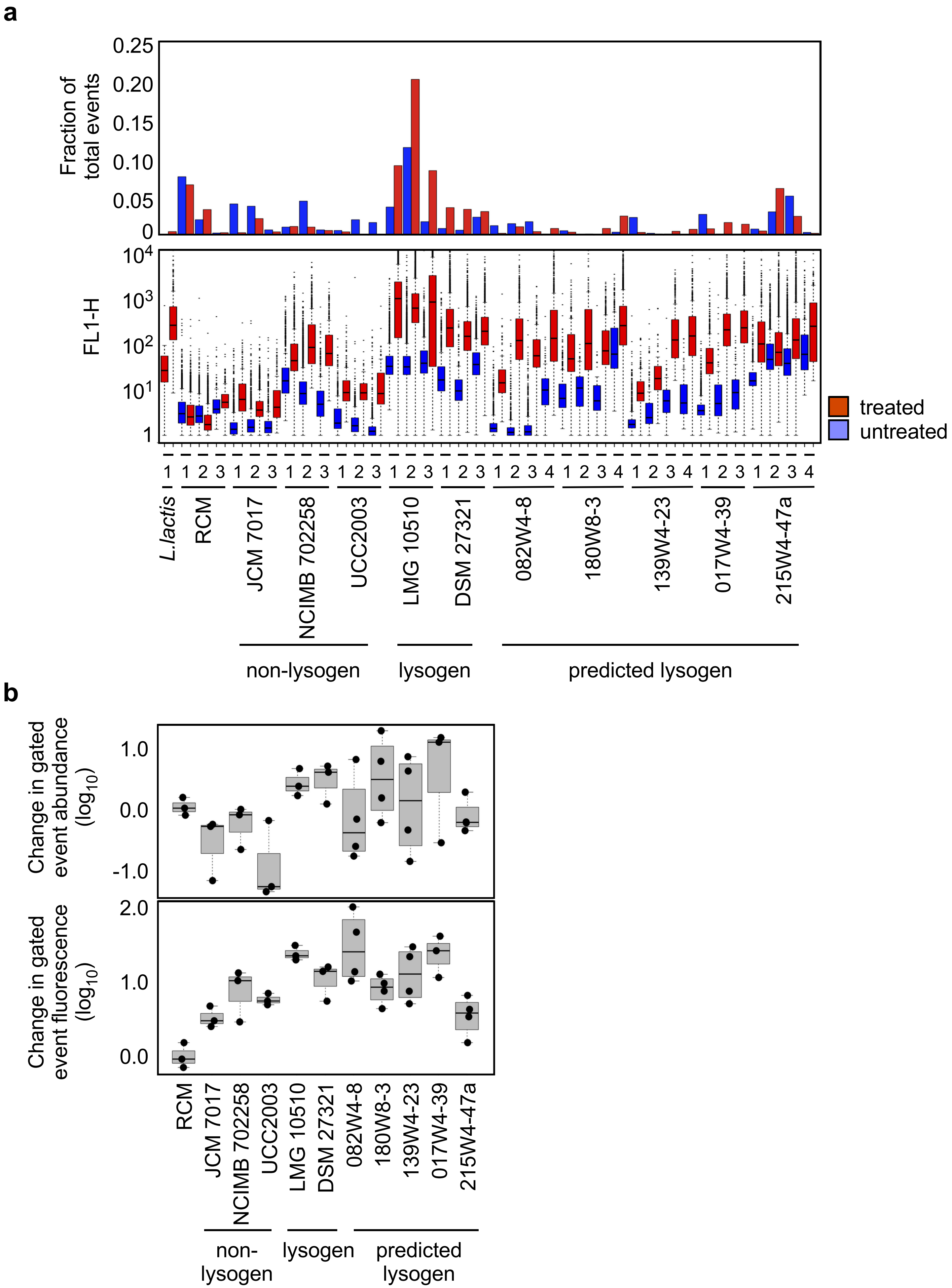

Supplementary Figure S5

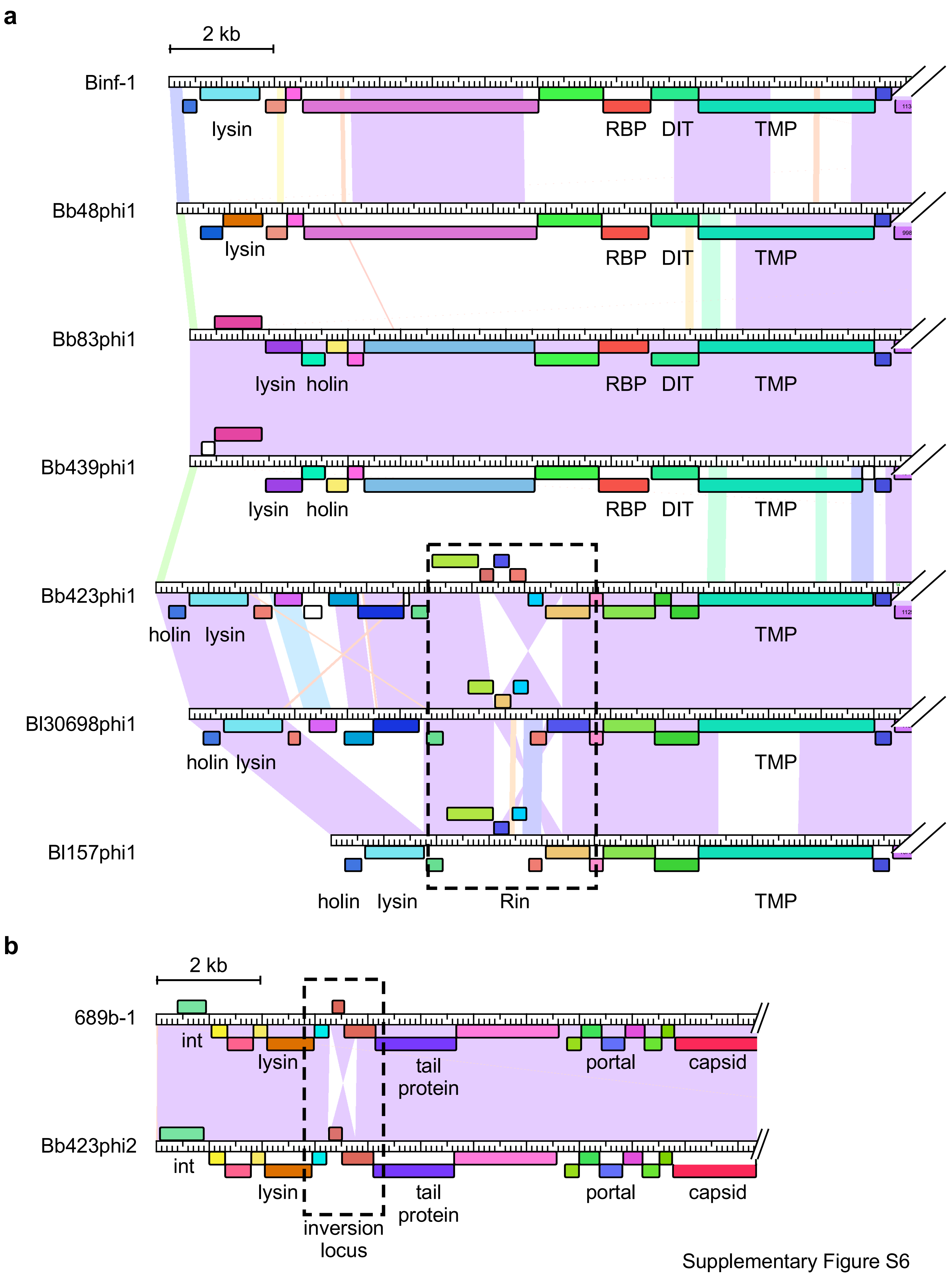

Supplementary Figure S6

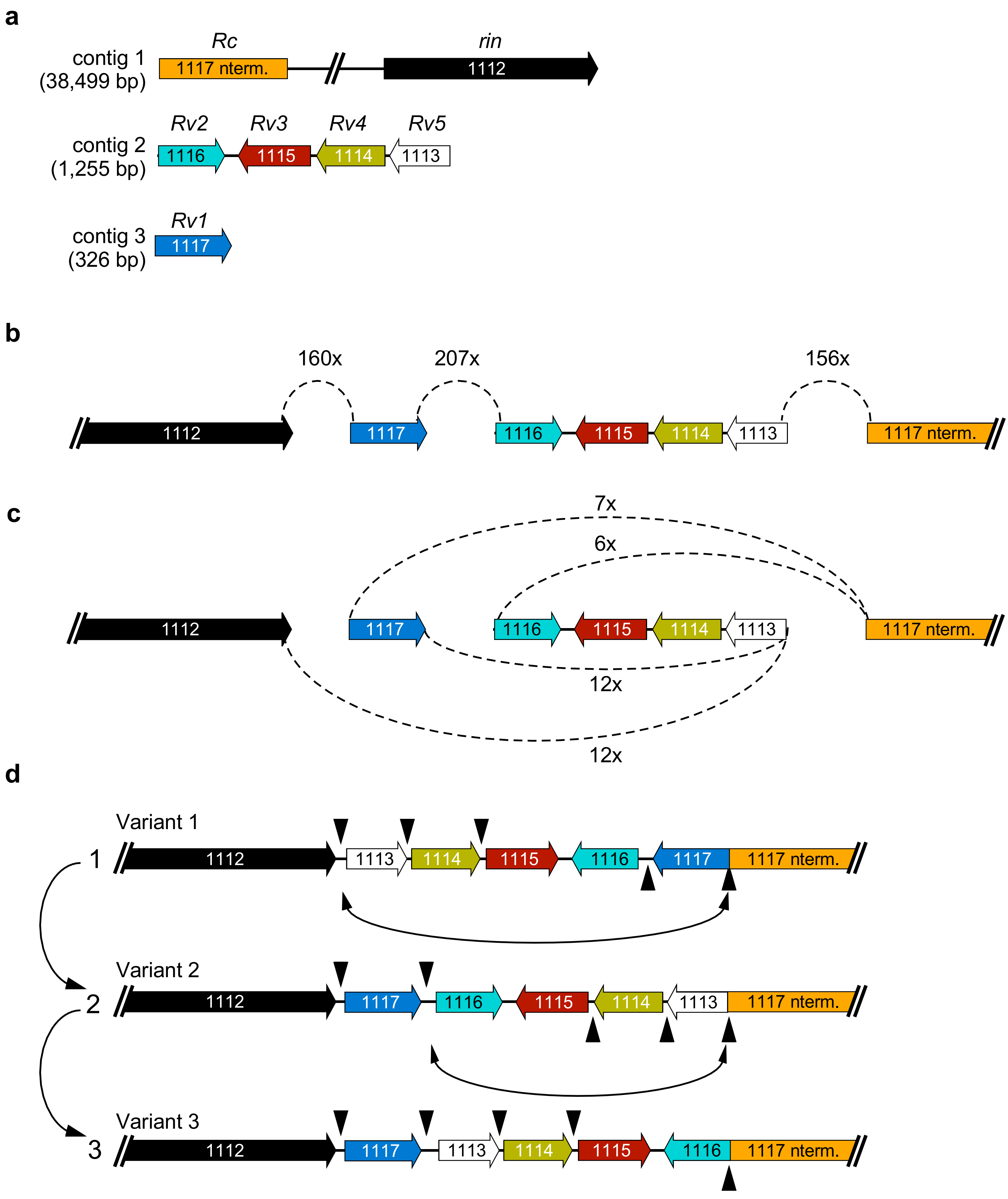

Supplementary Figure S7

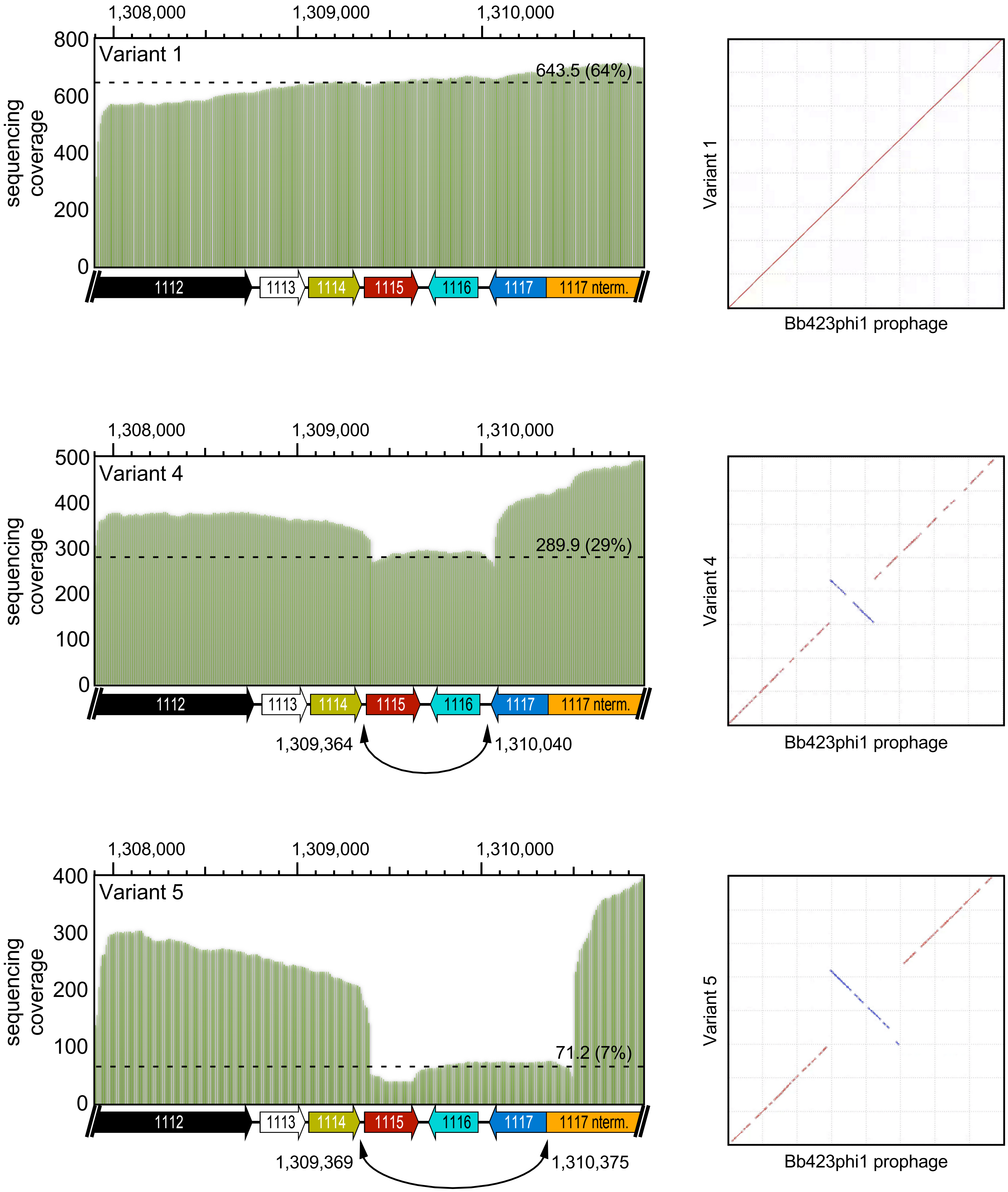

Supplementary Figure S8

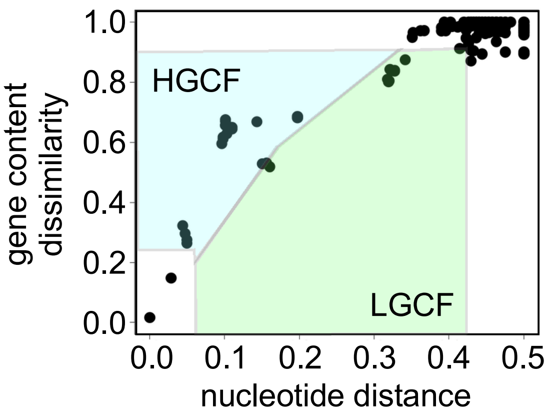

Supplementary Figure S9
